# Polycomb repression works without Siesta, the *Drosophila* ortholog of mammalian PCGF3

**DOI:** 10.1126/sciadv.aec0733

**Published:** 2026-03-06

**Authors:** Tatyana G. Kahn, Andres Garrido, Anastasiya Yushkova, Maria Kim, Alexander Glotov, Sweda Sreekumar, Jan Larsson, Yuri B. Schwartz

**Affiliations:** Department of Molecular Biology, Epigenetic Cooperation North (EpiCoN), Umeå University, 901 87 Umeå, Sweden.

## Abstract

Polycomb group proteins mediate epigenetic repression via multisubunit complexes, including canonical Polycomb Repressive Complex 1 (PRC1), which monoubiquitylates histone H2A and binds histone H3 trimethylated at lysine-27 (H3K27me3). The RING1 subunit of PRC1, critical for H2A ubiquitylation, forms other complexes. These variant RING1 complexes also ubiquitylate H2A but cannot bind H3K27me3, and their role in epigenetic repression is debated. Using *Drosophila* genetics, we found that canonical PRC1 and variant RING1 complexes ubiquitylate H2A at distinct genomic regions. We established that the *Drosophila* PCGF protein specific for variant RING1 complexes, which we named Siesta, is not required for epigenetic repression of developmental genes but controls larval locomotion independently of H2A ubiquitylation. Leveraging a massively parallel transgenic approach, we demonstrated that H2A ubiquitylation has minimal impact on transcriptional repression. Our findings imply that Siesta-RING1 complexes operate outside the Polycomb regulatory system and that the popular PRC1 classification will benefit from revision.

## Teaser

The study of the *Drosophila* PCGF3 ortholog revisits the PRC1 taxonomy and questions the role of H2AK118 ubiquitylation.

## INTRODUCTION

Polycomb group proteins are well-known for epigenetic repression of specific master-regulatory genes, thereby ensuring that cells maintain their developmental identities. These proteins act as multisubunit complexes, traditionally classified into two families: Polycomb Repressive Complex 1 (PRC1) and Polycomb Repressive Complex 2 (PRC2). The originally characterized PRC1 complex (purified from *Drosophila melanogaster* cells and sometimes referred to as canonical) consisted of Polycomb (Pc), Polyhomeotic (Ph), Sex combs on midleg (Scm) proteins, as well as the heterodimer between RING1 [the product of *Sex combs extra* (*Sce*) gene] and Posterior sex combs (Psc) proteins (*1*). The Pc subunit of the canonical PRC1 specifically binds to histone H3 trimethylated at lysine-27 (H3K27me3) (*2*), a modification produced by PRC2 complexes (*3*, *4*). Genes repressed by the Polycomb system are embedded in broad chromatin domains enriched in H3K27me3 (*5*, *6*). This histone modification acts as a molecular mark that helps to propagate the repressed state following DNA replication (*7*, *8*).

Subsequent proteome analyses of RING1 partners in mouse and human cells (mammals have two closely related orthologs RING1 and RING2 encoded by the *RING1* and *RNF2* genes) revealed not only complexes analogous to canonical PRC1 but also a series of complexes lacking orthologs of Pc, Ph, and Psc (*9*–*11*). These variant complexes centered around the heterodimer between RING1/2 orthologs and one of the four PCGF proteins (PCGF1, PCGF3, PCGF5, or PCGF6). These RING1/2 complexes incorporate RYBP (or a closely related YAF2 protein) instead of Pc orthologs. They also include additional subunits absent from canonical PRC1 that vary depending on the identity of the PCGF subunit.

Variant RING1/2 complexes, sometimes referred to as noncanonical PRC1, were initially thought to have evolved from ancestral canonical PRC1, presumably via the expansion of the PCGF protein family. However, systematic comparison of the genomes from diverse animal clades has shown that the entire complement of the *PCGF* genes found in vertebrates appeared early in animal evolution (*12*). Some of these genes were subsequently lost in specific lineages, including *D. melanogaster* (*12*). Genetic studies in *D. melanogaster* (hereafter referred to as *Drosophila*) and mice leave no doubt that the canonical PRC1, which first emerged in the metazoan lineage (*12*, *13*), is critical for the repression of developmental genes (*14*–*19*). In contrast, the role of variant metazoan RING1/2 complexes remains less well defined. Given that these complexes did not evolve from canonical PRC1, it cannot be assumed that all variant RING1/2 assemblies function as epigenetic transcriptional repressors.

Functional studies of variant RING1/2 complexes provide mixed insights. In the developing mouse neocortex, genetic disruption of canonical PRC1 impairs the maintenance of cell lineage identity during neurogenesis and gliogenesis, whereas variant RING1/2 complexes appear to play a minor role (*20*). Conversely, studies in mouse embryonic stem cells presented conflicting evidence. Some suggested that variant RING1/2 complexes synergize and are functionally more important than canonical PRC1 to repress developmental genes (*21*). Others reported that canonical PRC1 plays a central role (*22*). Furthermore, in mouse brain cells, RING2-PCGF3 complexes bind in the vicinity of transcription start sites (TSSs) of a subset of active genes, where they appear to promote rather than repress transcription (*23*, *24*).

To what extent the catalytic activity of RING1 complexes is important for the repression of developmental genes is another open question. Both canonical PRC1 and variant RING1/RING2 complexes function as E3 ligases, monoubiquitylating histone H2A at a conserved residue, lysine-119 in mammals (H2AK119ub) and lysine-118 in *Drosophila* (H2AK118ub) (*9*, *25*, *26*). Variant RING1/RING2 complexes seem to be more active E3 ligases in vitro compared to canonical PRC1 (*27*). This may be due to the differences in the biochemical properties of distinct PCGF proteins (*28*) or additional stimulation by the RYBP/YAF2 subunit, which is unique to the variant complexes (*9*, *27*). With a size of ~8.5 kDa, ubiquitin may physically affect chromatin structure. Thus, single-molecule magnetic tweezers experiments suggest that H2AK119ub stabilizes in vitro reconstituted nucleosome particles by preventing the DNA unwrapping from the histone core (*29*). This may inhibit transcription. On the other hand, fluorescence resonance energy transfer measurements suggest that H2AK119ub interferes with chromatin fiber folding (*30*), making DNA more accessible to transcription regulators. In addition, H2AK119ub increases the affinity of PRC2 complexes containing accessory subunits Aebep2 and Jarid2, which, in turn, stimulates H3K27 methylation by this PRC2 variant (*31*, *32*).

Consistent with its potential involvement in repression, H2AK119ub (and H2AK118ub in flies) is enriched within developmental genes repressed by the Polycomb system (*21*, *30*, *33*–*36*). In mouse embryonic stem cells, the replacement of RING1 and RING2 with a variant lacking E3 ligase activity led to a loss of H2AK119ub and a correlating increase in transcription of many developmental genes normally repressed by the Polycomb system (*33*, *36*). However, the mechanistic link between the loss of H2AK119ub and derepression remains ambiguous as these cells also exhibited a loss of canonical PRC1, PRC2, and H3K27me3 from the same genes, making it difficult to isolate the specific contribution of H2A ubiquitylation. Notably, *Drosophila* mutants in which histone H2A is replaced by a truncated version carrying lysine-to-arginine substitutions at positions 117, 118, 121, and 122, rendering it nonubiquitylatable by RING1 complexes, retain repression of homeotic selector genes (*37*). This argues that, even if H2AK119/K118 ubiquitylation contributes to repression in some species or cell types, it is unlikely to represent a universal mechanism by which the Polycomb system represses developmental genes.

**Fig. 1.**
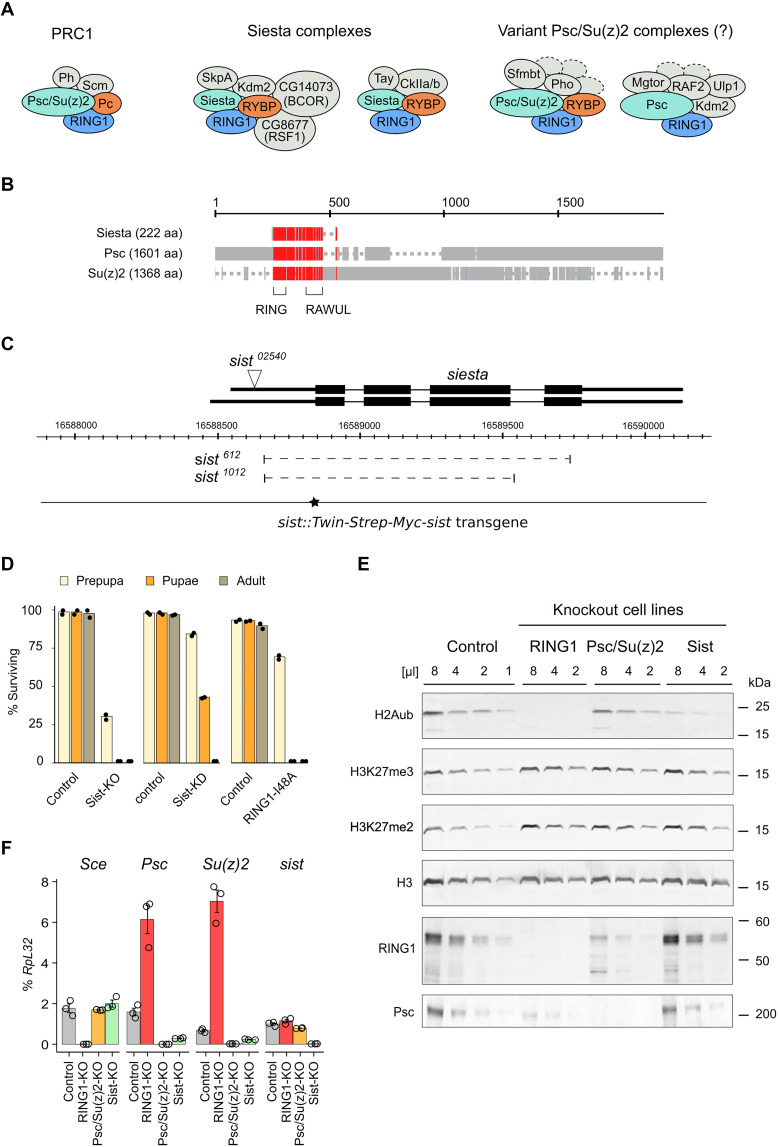
*siesta* alleles and the interrelation between PRC1 and Siesta-RING1 complexes. (**A**) RING1 complexes of *D. melanogaster*. (**B**) Constraint-based alignment (*94*) of *Drosophila* PCGF proteins. Highly conserved positions are indicated in red, positions with lower conservation are in blue, and nonconserved positions are in gray. Gaps in the alignment are shown as dashed lines. The positions of RING and RAWUL domains are shown below, and the coordinate scale [in amino acids (aa)] are above the aligned sequences. (**C**) Schematics of the *siesta* locus. Two alternative transcripts above the coordinate scale (*dm6* genomic release) are shown with the TSS to the left. Thin lines indicate introns, and black boxes are the coding parts. The position of transposon insertion in the *sist^02540^* allele is indicated by a white triangle. Dashed lines mark the extent of *sist^612^* and *sist^1012^* deletions, and the solid line marks the extent of the *sist::Twin-Strep-Myc-sist* transgene (black star indicates the position of Twin-Strep and Myc tags) sufficient to complement the *sist* loss-of-function mutations. (**D**) Lethal stage of Siesta-KO (*sist^612^*/*sist^1012^*), Siesta-KD (*sist^612^*/*sist^02540^*), and RING1-I48A (*sce^I48A^*/*sce^I48A^*) mutants. Matching heterozygous controls were used for comparison. The bar plots indicate the average survival rate, starting with 150 first instar larvae. The black dots show the results of two independent experiments. (**E**) Western blots with twofold dilutions of total nuclear protein from the control, RING1-KO, Psc/Su(z)2-KO, and Siesta-KO cell lines probed with antibodies indicated to the left. Positions of molecular weight markers (in kilodaltons) are shown to the right. (**F**) Relative transcript abundance for the *Sce*, *Psc*, *Su(z)2*, and *sist* genes in the control, RING1-KO (the cell line lacking the *Sce* gene), Psc/Su(z)2-KO, and Siesta-KO cells. The bar plots show the average of three independent RT-qPCR experiments performed with three independently prepared RNA samples. Circles show results of individual experiments, and whiskers indicate the SEM.

Comparing how the epigenetic repression of developmental genes is affected by genetic ablation of variant RING1/RING2 complexes or canonical PRC1 may help to understand their relative contributions and that of the H2AK119 ubiquitylation. Removing individual PCGF proteins would be one way to accomplish this. Such studies are notoriously difficult to conduct in mammalian species because they have six PCGF proteins, all encoded by separate genes located in different parts of the genome. The *Drosophila* model offers a powerful alternative to overcome this complexity. The *Drosophila* genome encodes just three PCGF proteins. Two of them are products of adjacent, closely related *Psc* and *Su(z)2* genes (*38*), which arose through lineage-specific duplication (*12*). The corresponding Psc and Su(z)2 proteins are incorporated into the canonical PRC1 (Fig. 1, A and B) (*1*, *39*). The third PCGF protein, encoded by the gene previously known as *l(3)73Ah*, which we propose to rename as *siesta*, is an ortholog of mammalian PCGF3. A recent proteomics study indicates that Siesta protein is incorporated in two kinds of RING1 complexes (*40*). One kind contains the RYBP, SkpA, Kdm2, and BCOR proteins (Fig. 1A) and corresponds to the mammalian variant RING1/2 complex incorporating PCGF1. Another kind contains the RYBP, Tay, and CkIIa/b proteins (Fig. 1A) and corresponds to the mammalian variant RING1/2 complexes incorporating either PCGF3 or PCGF5. In other words, although Siesta has evolved from a common ancestor with mammalian PCGF3, it appears functionally analogous to mammalian PCGF1, PCGF3, and PCGF5.

In addition to canonical PRC1, the Psc and Su(z)2 proteins may be incorporated into variant RING1 complexes. However, the composition and nature of these complexes is not entirely clear. Lagarou and colleagues reported a RING1 complex consisting of Psc, RING1, Kdm2, Ulp1, RAF2, and Mgtor (Fig. 1A) that could be immunopurified from embryonic nuclear extracts depleted of the Pc protein before purification (*41*). Yet, a more recent cross-linking and tandem affinity purification failed to detect this complex or find evidence that Kdm2 is incorporated into RING1 complexes lacking RYBP and Siesta (*40*). Instead, the cross-linking and tandem affinity purification procedure suggests interactions between Psc/Su(z)2 and RING1, RYBP, Sfmbt, and Pleiohomeotic (Pho) (Fig. 1A) (*40*). Whether these interactions represent a distinct protein complex or relate to multiple biochemical processes [e.g., interactions between canonical PRC1 and Pho-Repressive Complex (PhoRC) (*42*, *43*) and tripartite interactions between Psc/Su(z)2, RING1, and RYBP] remains to be investigated.

Here, we leveraged the power of *Drosophila* genetics to find that canonical PRC1 and Siesta-RING1 complexes monoubiquitylate H2AK118 across distinct genomic regions. We found that Siesta-RING1 complexes control larval locomotion independently of H2AK118 ubiquitylation and are dispensable for the epigenetic repression of *Drosophila* homeotic genes. Exploiting the division of labor between PRC1 and Siesta-RING1 complexes, we used thousands of reporters integrated in parallel to conclude that H2AK118 ubiquitylation, by itself, is unlikely to have a major repressive effect on transcription.

## RESULTS

To clarify the contribution of PRC1 and variant RING1 complexes to H2AK118 monoubiquitylation and the repression by the Polycomb system, we turned to the two loss-of-function alleles of the *l(3)73Ah* gene, which we briefly mentioned in the earlier study (*40*). The PCGF protein encoded by the *l(3)73Ah* gene is an ortholog of mammalian PCGF3. The loss-of-function alleles of the *l(3)73Ah* gene generated by CRISPR-Cas9–mediated genome editing contain 1052– and 868–base pair (bp) deletions that remove the start codon and almost the entire open reading frame (ORF) of the gene (Fig. 1C and fig. S1). Animals trans-heterozygous for the two alleles die during pupation (Fig. 1D), consistent with the reported phenotype of the original (now lost) ethyl methanesulfonate (EMS)–induced mutant allele (*44*, *45*). Normally, *Drosophila* larvae continuously forage for food as they have a limited time to gain the weight required to undergo metamorphosis (*46*). In contrast, the trans-heterozygous mutant larvae display an obvious locomotion defect (described in detail in a later section), which manifests in prolonged periods of inactivity between bouts of crawling.

The discoverers gave the original *l(3)73Ah* gene name to mark the gene’s position relative to other complementation groups identified in their mutagenic screen (*44*). This name has no connection to the gene function and is hard to remember, pronounce, and write. Following the long-standing tradition of naming *Drosophila* genes by their visible mutant phenotype, we propose to rename *l(3)73Ah* to *siesta* (*sist*) and will refer to the gene and its product as such hereafter. In line with the proposed nomenclature, we will refer to the loss-of-function alleles as *sist^612^* [formerly *l(3)73Ah^612^*] and *sist^1012^* [formerly *l(3)73Ah^1012^*].

### Multiple factors control the relative abundance of Psc/Su(z)2-RING1 and Siesta-RING1 complexes

We reasoned that a smaller repertoire of *Drosophila* PCGF proteins could provide a clearer picture of the genomic distribution and extent of H2AK118 ubiquitylation mediated by various RING1 complexes. To avoid the problem of maternal contribution and to have unrestricted amounts of material, we derived a cultured cell line from *Drosophila* embryos homozygous for the *sist^612^* allele using the *Ras*^*V12*^ transformation approach (*47*). Although *sist* is essential for viability, the mutant cells (hereafter referred to as Siesta-KO) are viable and proliferate in culture. Consistent with previous RNA interference (RNAi) knockdown studies (*34*), Siesta-KO cells lose ~80% of the bulk H2AK118ub, detected in the control *Ras*^*V12*^ transformed but otherwise wild-type cells (Fig. 1E). This is a substantially greater reduction of the bulk H2AK118ub compared to ~20% loss seen in Psc/Su(z)2-KO cells (Fig. 1E). To ensure that the antibodies used to assay the H2AK118ub are specific, we edited the genome of *Ras*^*V12*^ transformed control cells (see Materials and Methods section for details) and derived a cultured cell line where the *Sce* gene encoding the RING1 protein is deleted. In these cells (hereafter referred to as RING1-KO), Western blot analysis detects no trace of H2AK118ub, affirming that our assay is highly specific (Fig. 1E). Although H2AK119ub was shown to stimulate the catalytic activity of human PRC2 in vitro (*31*, *32*), we see no obvious reduction of the bulk di- or trimethylated H3K27 in Siesta-KO or RING1-KO cells (Fig. 1E).

The relation between intranuclear levels of RING1, Psc, and Siesta appears more complicated. As expected, RING1 is undetectable in RING1-KO cells, and Psc is absent in Psc/Su(z)2-KO cells (Fig. 1E). Unexpectedly, we observed a substantially (~4 times) lower amount of RING1 in Psc/Su(z)2-KO cells and, conversely, ~4 times less Psc in RING1-KO cells (Fig. 1E). On the other hand, the differences in the levels of RING1 and Psc in Siesta-KO cells, if any, were too small to be reliably detected (Fig. 1E). As no antibodies against Siesta are currently available, we could not track its levels in our cultured cell lines. The reverse transcription quantitative polymerase chain reaction (RT-qPCR) analysis indicated that transcription of the *Sce* gene is the same in the control, Psc/Su(z)2-KO, and Siesta-KO cells (Fig. 1F), indicating that the reduction in RING1 protein abundance occurs posttranscriptionally. Although we cannot formally exclude that Psc/Su(z)2-KO and Siesta-KO are needed for the efficient translation of RING1 mRNA, it is more plausible that RING1 becomes unstable when not in the complex with Psc, Su(z)2, or Siesta. Given that Psc/Su(z)2-KO has a more pronounced effect on nuclear RING1 pool than Siesta-KO, our data suggest that most RING1 resides in the complex with Psc/Su(z)2.

The effects of RING1 and Siesta knockout on the nuclear Psc pool are even more interesting. *Psc* and *Su(z)2* genes are situated next to each other. The gene cluster includes multiple Polycomb response elements (PREs) bound by PRC1 and PRC2 (*6*, *48*), resulting in a feedback loop where mutations in genes encoding for PRC1 subunits increase *Psc* and *Su(z)2* transcription (*49*). In agreement with this, *Psc* and *Su(z)2* transcription in RING1-KO cells increases 4- to 10-fold (Fig. 1F). Despite this, the amount of Psc protein in RING1-KO cells is notably lower than that in control cells, suggesting that Psc is unstable in the absence of RING1 and that elevated transcription of the *Psc* gene cannot compensate for this. Curiously, in Siesta-KO cells, transcription of the *Psc* and *Su(z)2* genes is reduced (Fig. 1F). No anti-Su(z)2 antibodies are currently available, so we could not track it in our experiments. We posit that, in *Drosophila* cells, the amount of RING1 is limited. In the absence of Siesta, more RING1 protein is available for incorporation into PRC1, which, in turn, increases the intranuclear concentration of PRC1 and leads to stronger transcriptional repression of the *Psc-Su(z)2* locus. Such a feedback loop may have evolved to keep the appropriate balance between PRC1 and Siesta complexes sharing the common RING1 subunit.

To conclude, our observations indicate that, in *Drosophila* cells, most of the RING1 protein resides in complexes with Psc/Su(z)2. Yet, it is the comparatively less abundant Siesta-RING1 complexes that are responsible for generating the bulk of steady-state H2AK118ub.

### Psc/Su(z)2-RING1 and Siesta-RING1 complexes ubiquitylate H2AK118 in different parts of the genome

**Fig. 2.**
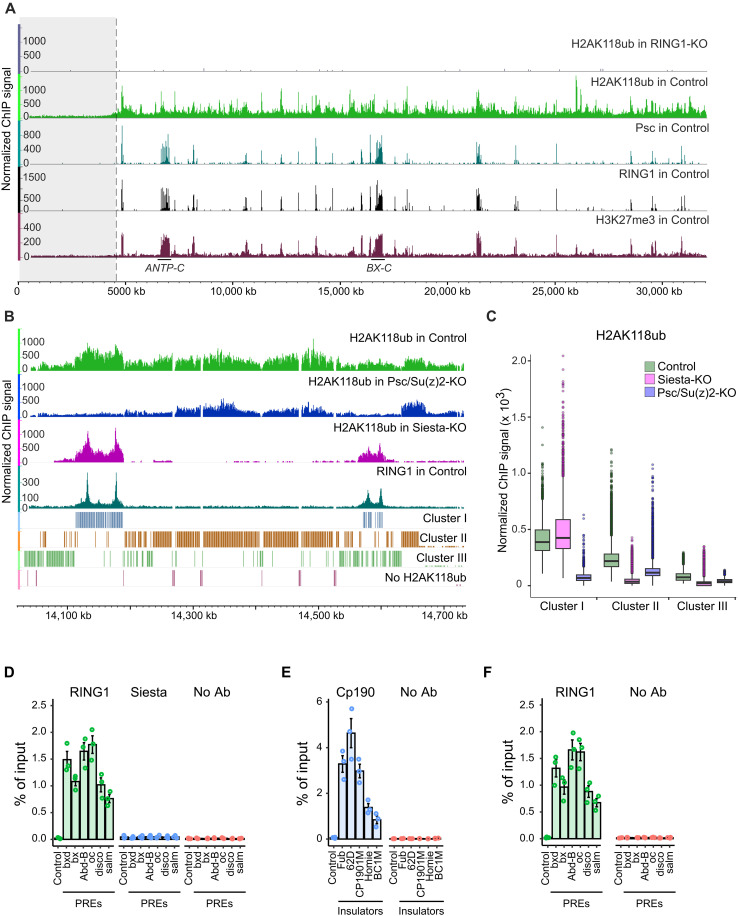
Genome-wide H2AK118 ubiquitylation by Psc/Su(z)2-RING1 and Siesta-RING1 complexes. (**A**) Genome browser tracks of H2AK118ub ChIP-seq signals in RING1-KO and control cell lines across chromosome 3R. Tracks for Psc, RING1, and H3K27me3 in control cells help to pinpoint genes repressed by the Polycomb system. The location of *ANTP-C* and *BX-C* is indicated above the coordinate scale (*dm6* genome release). The shaded region marks the extent of pericentromeric heterochromatin (*52*). (**B**) ChIP-seq signals for H2AK118ub and RING1 in control, Psc/Su(z)2-KO, and Siesta-KO cell lines across a representative region of chromosome 3L. The genome segmentation into 1-kb regions of four different categories is shown below the ChIP-seq tracks. (**C**) H2AK118ub ChIP-seq signals in 1-kb genomic regions of different categories in control, Siesta-KO, and Psc/Su(z)2-KO cells. Note the strong H2AK118ub loss within Cluster I regions in Psc/Su(z)2-KO cells and the H2AK118ub drop within Cluster II and III regions in Siesta-KO cells. Box plots indicate medians and span the interquartile range with whiskers extending 1.5 times the range. Dots designate the outliers. Pairwise comparisons between the datasets indicates that, for all pairs, the values have significantly different distributions (Wilcoxon rank sum test, *P* value < 2.2 × 10^−16^). (**D**) ChIP-qPCR with chromatin prepared from embryos expressing the Twin-Strep–tagged Siesta protein. Note the robust immunoprecipitation of PREs with antibodies (Ab) against RING1 but not with Strep-Tactin resin, specific for tagged Siesta protein. Here and in (E) and (F), the bar plots show the average of three independent ChIP-qPCR experiments performed with three independently prepared chromatin samples. Circles show results of individual experiments, and whiskers indicate the SEM. (**E** and **F**) Control experiments with chromatin lysates from embryos expressing the Twin-Strep–tagged Cp190 protein. Here, insulator elements (*95*) display immunoprecipitation with Strep-Tactin resin, specific for tagged Cp190 (E), and PREs (*34*) display immunoprecipitation with anti-RING1 antibodies (F).

Sequencing of DNA immunoprecipitated with anti-H2AK118ub antibodies from chromatin lysates of control cells (H2AK118ub ChIP-seq) revealed a broad distribution of the chromatin immunoprecipitation sequencing (ChIP-seq) signal throughout the genome (Fig. 2A). In contrast, H2AK118ub ChIP-seq with chromatin lysates from RING1-KO cells resulted in a uniform and low signal across the genome, confirming that our assay is accurate and specific (Fig. 2A). To enable quantitative comparison of ChIP-seq signals in cells with different genomic backgrounds, we adopted the “sans-spike-in” approach for quantitative ChIP-seq (siQ-ChIP) proposed by Dickson and colleagues (*50*, *51*). This approach is straightforward and avoids potential pitfalls associated with adding small amounts of chromatin from other species to the immunoprecipitation reaction (so-called “spike-in” approach) that is often used for ChIP-seq signal normalization (*51*). Briefly, all ChIP-seq assays were performed with a fixed amount of cross-linked chromatin lysates and the same amount of a given antibody. For each sample, we recorded the fraction of immunoprecipitated DNA that was used for sequencing. The values were then used for scaling ChIP-seq signals to represent total ChIP yields (i.e., the signal expected if the entire immunoprecipitated DNA has been sequenced), adjusted for the sequencing depth (see the Materials and Methods section for a detailed description of the procedure). We performed two independent ChIP-seq experiments for each cell line and antibody and sequenced the DNA from the corresponding chromatin input materials to control for potential sample processing and sequence alignment biases. The genomic distributions of ChIP-seq signals for the corresponding replicate experiments are highly concordant (*r* = 0.90 for control cells and 0.74 for RING1-KO cells; also see fig. S2A and table S1).

A side-by-side comparison of H2AK118ub ChIP-seq signals, averaged over 1-kb genomic windows, for the control and RING1-KO cells indicates that 82% of the annotated *Drosophila* genome exhibit detectable levels of H2AK118ub. In most of the windows (78%) where the H2AK118ub was not detected, this happened because the windows contained repeated DNA and could not be uniquely matched to the sequencing reads. The remaining windows with unique nucleotide sequences but no detectable H2AK118ub are scattered throughout the genome, with notably higher density within pericentromeric regions. These regions, sometimes referred to as pericentromeric heterochromatin, are decorated with the HP1 protein and nucleosomes di- and trimethylated at lysine-9 of histone H3 (*52*). Even excluding the windows without detectable H2AK118ub, the windows corresponding to pericentromeric heterochromatin display substantially lower H2AK118ub ChIP-seq signals compared to the rest of the genome (Fig. 2A and fig. S2, B and C). A similar observation was made during H2AK118ub profiling in spermatocytes and wing imaginal disc cells (*53*). The low H2AK118ub in the pericentromeric regions may be common for many cell types due to HP1-mediated changes in chromatin structure that hinder the action of RING1 complexes.

To understand the contribution of Psc/Su(z)2-RING1 and Siesta-RING1 complexes to the H2AK118 ubiquitylation throughout the genome, we mapped the H2AK118ub distribution in Siesta-KO and Psc/Su(z)2-KO (*34*) cells. We supplemented these experiments with ChIP-seq mapping of H3K27me3, Psc and RING1 in the control cells, which revealed sites where RING1 complexes are bound stably and marked the genes repressed by the Polycomb system, including positions of associated PREs. We also mapped RING1 in the RING1-KO cells to confirm the specificity of the anti-RING1 antibody. Visual inspection of ChIP-seq signals shows that peaks of the RING1 ChIP-seq signal (the sites of stable RING1 binding) are confined to peaks of Psc signal embedded in stretches of chromatin enriched in H3K27me3 (Fig. 2A and fig. S2A). This indicates that PREs are the only sites in the *Drosophila* genome stably bound by RING1 complexes. In line with this suggestion, stretches of chromatin around PREs with elevated ChIP-seq signals for H3K27me3 typically show elevated signals for H2AK118ub (Fig. 2A and fig. S2A). However, there are notable exceptions. As pointed out earlier, the chromatin of the bithorax complex (*BX-C*) and Antennapedia (*ANTP-C*) homeotic gene clusters contains very little H2AK118ub (*30*, *35*), despite these genes being repressed and their PREs bound by RING1 (fig. S3). The systematic search for PRE-equipped loci with elevated H3K27me3 but low H2AK118ub revealed two similar cases: β *amyloid protein precursor-like* (*Appl)*–*ventral nervous system defective* (*vnd)* locus and *Visual system homeobox 1 (Vsx1)*–*Visual system homeobox 2 (Vsx2)* gene cluster (figs. S3 and S4). Products of these four genes control aspects of nervous system development, and *vnd*, *Vsx1*, and *Vsx2* encode homeobox transcription regulators (*54*–*56*). As suggested by Bonnet and colleagues, the low steady-state levels of H2AK118ub at these loci may be explained by an exceptionally efficient deubiquitylation of H2AK118ub by the PRE-bound PR-DUB complex (*30*). We posit that mutations in genes encoding PR-DUB subunits may have a particularly strong negative impact on the epigenetic repression of *Appl*, *vnd*, *Vsx1*, and *Vsx2*, similar to that observed for homeotic genes of the bithorax complex (*57*).

The H2AK118ub ChIP-seq signal at PRE-equipped loci is markedly reduced in Psc/Su(z)2-KO cells but remains unaffected or even slightly increased in Siesta-KO cells (Fig. 2B). This argues that H2AK118ub at PRE-equipped loci is catalyzed by Psc/Su(z)2-containing RING1 complexes, most likely canonical PRC1. Further strengthening this conclusion, we detected no Twin-Strep-tag–mediated immunoprecipitation of PREs DNA out of cross-linked chromatin lysates prepared from embryos where the loss of endogenous *siesta* function was complemented by transgenic expression of tagged Siesta protein (*sist::Twin-Strep-Myc-sist*; *sist^612^*/*sist^1012^* embryos) (Figs. 1C and 2, D to F). This indicates that, in contrast to PRC1, Siesta-RING1 complexes are not attracted to PREs.

As estimated previously (*58*), regions surrounding PREs that are highly enriched in H3K27me3 and decorated by PRC1-dependent H2AK118ub account for only ~5% of the *Drosophila* genome. Most of the remaining genome also shows significantly elevated H2AK118ub ChIP-seq signal, with some of the sites displaying signals comparable in magnitude to those seen at PRE-equipped loci (Fig. 2A and fig. S2A). None of these sites display elevated ChIP-seq signals for RING1, suggesting that H2AK118 ubiquitylation outside PRE-equipped loci is installed without stable binding of RING1 complexes. This widespread H2AK118 ubiquitylation is strongly reduced in Siesta-KO cells (Fig. 2B and fig. S2A). The effect is not limited to cultured cells. The immunostaining of polytene chromosomes from the *sist^02540^*/*sist^612^* (Sist-KD/KO) third instar larvae shows equally marked loss of H2AK118ub throughout chromosome arms except at PRE-equipped loci, most brightly stained with antibodies against H3K27me3 (fig. S2, D and E).

Unsupervised *k*-means clustering of 1-kb genomic segments (bins) based on comparison of H2AK118ub ChIP-seq signals in the control, Psc/Su(z)2-KO, and Siesta-KO cells reinforces the observations above. The clustering partitions the genome into regions of three kinds: PRE-equipped loci with high H2AK118ub dependent on Psc/Su(z)2 complexes (Cluster I), regions with high H2A118ub strongly dependent on Siesta-RING1 complexes (Cluster II), and regions with low but significant H2A118ub also primarily dependent on Siesta-RING1 complexes (Cluster III) (Fig. 2, B and C). Completing the segmentation of the genome are regions devoid of H2AK118ub discussed above and excluded from the unsupervised *k*-means clustering procedure (Fig. 2B and fig. S5). Curiously, a side-by-side comparison of H2AK118ub ChIP-seq signals in Cluster II and Cluster III regions in Psc/Su(z)2-KO and control cells reveals a small but detectable reduction of the signal upon Psc/Su(z)2 loss (Fig. 2C). This argues for modest hit-and-run H2AK118 ubiquitylation activity of untethered PRC1 or Psc/Su(z)2-containing variant RING1 complexes genome-wide.

To summarize, our observations indicate that PREs are the only sites in the *Drosophila* genome stably bound by RING1 complexes. These complexes, most likely canonical PRC1, contain Psc or Su(z)2 proteins and produce most of the H2AK118ub at PRE-regulated genes. At the same time, the hit-and-run action of Siesta-RING1 complexes is responsible for the bulk of H2A118ub elsewhere in the genome.

### Siesta complexes control larval locomotion independently of H2AK118 ubiquitylation

Trans-heterozygous *sist^612^*/*sist^1012^* zygotic null larvae die during pupation. They do not exhibit homeotic transformations but display a pronounced locomotion defect characterized by reduced crawling speed. This phenotype is distinct from that of the mutants for genes encoding canonical PRC1 subunits. The *sist^612^*/*sist^1012^* mutant animals become viable, fertile, and can be maintained as a stock when supplemented with the transgene containing 2.5-kb fragment of genomic DNA that encompasses the entire *siesta* transcript preceded by the short Twin-Strep-Myc tag, plus 613 bp of the DNA upstream of the TSS and 129 bp downstream of the transcript end (*sist::Twin-Strep-Myc-sist* transgene; Fig. 1C). The *sist::Twin-Strep-Myc-sist*; *sist^612^*/*sist^1012^* larvae crawl with the same speed as those of the common wild-type strain *Oregon-R* (fig. S6). This ensures that the lethality and the locomotion defect are caused by the disruption of *siesta* function and not due to a second-site mutation.

Given that Siesta-RING1 complexes monoubiquitylate H2AK118 outside the genes controlled by PRC1, we asked whether the locomotion defects observed in *sist* mutants are linked to the widespread depletion of H2AK118ub across the genome. To this end, we tracked the locomotion of *sist^612^*/*sist^1012^* (Siesta-KO) second instar larvae and compared them to the locomotion of the *sist^612^*/*sist^02540^* (Siesta-KD) and *Sce^I48A.gen^; Sce^KO^* (RING1-I48A) animals. The *sist^02540^* allele is the insertion of the *PZ* transposable element (*59*) in the 5′ untranslated region (5′UTR) of *sist* (Fig. 1C). As shown in Fig. 1D, *sist^612^*/*sist^02540^* animals survive longer than Siesta-KO, indicating that *sist^02540^* retains partial function and should be considered as a hypomorphic allele. The RING1-I48A animals are homozygous for the loss-of-function *Sce^KO^* allele (*60*) and carry a transgene expressing a catalytically impaired RING1 protein in which isoleucine-48 is substituted with alanine (fig. S7). This mutation disrupts RING1’s E3 ligase activity toward H2AK118 (*37*). Like Siesta-KO mutants, RING1-I48A animals die during pupation (Fig. 1D).

**Fig. 3.**
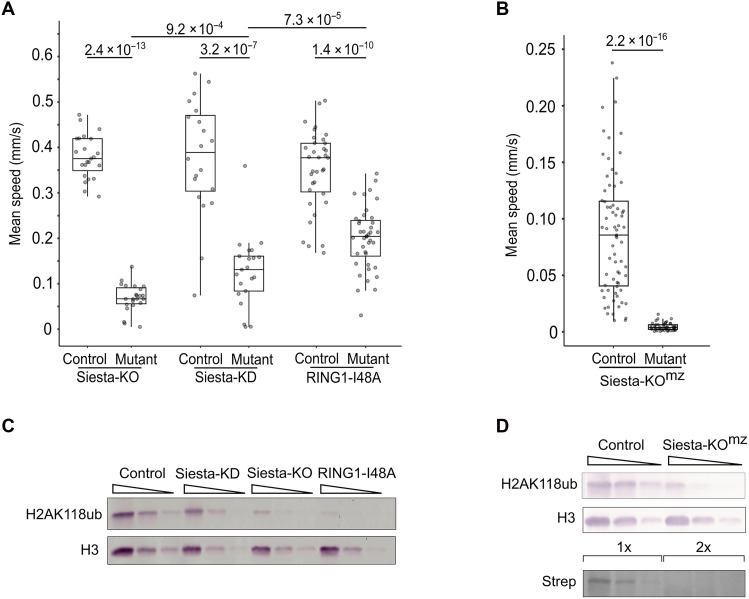
Siesta complexes control locomotion independently of the bulk H2AK118 ubiquitylation. (**A**) Motion tracking of Siesta-KO (*sist^612^/sist^1012^*), Siesta-KD (*sist^612^/sist^02540^*), and RING1-I48A (*Sce^I48A^; Sce^KO^*) second instar larvae. The RING1-I48A animals are homozygous for the loss-of-function *Sce^KO^* allele located on the third chromosome and have a transgene integrated into the second chromosome expressing a catalytically impaired RING1 protein in which isoleucine-48 is substituted with alanine. Heterozygous animals were used as matching controls. Here and in (B), the box plots show median speeds and span the interquartile range with whiskers extending 1.5 times the range. Gray dots indicate the mean speeds of individual larvae. The differences between the corresponding groups were tested for statistical significance using the Wilcoxon rank sum test, and *P* values are displayed above the box plots. (**B**) Motion tracking of Siesta-KO^mz^ and matching control first instar larvae. (**C**) Western blot analysis of H2AK118ub levels in various *siesta* mutants. Threefold serial dilutions of the total protein extract from second instar larvae were assayed using anti-H2AK118ub and pan-histone H3 (loading control) antibodies. (**D**) Threefold serial dilutions of the total protein extract from Siesta-KO^mz^ and control embryos, where the transgenic copy of *siesta* was not removed, were analyzed by Western blot with anti-H2AK118ub and anti-H3 antibodies. The Twin-Strep–tagged transgenic Siesta was detected using the streptavidin–alkaline phosphatase (AP) conjugate. Note that, in Siesta-KO^mz^ embryos, the transgenic Siesta protein is not detected even when twice as much protein extract is used compared to the control.

The Siesta-KO mutants move five times slower than heterozygous control larvae (Fig. 3A). The hypomorph Siesta-KD larvae move faster than Siesta-KO, and the RING1-I48A display the highest median speed of the three (Fig. 3A). Notably, these locomotion phenotypes did not correlate with the extent of bulk H2AK118ub loss (Fig. 3C). The latter is the most pronounced in the RING1-I48A larvae and the least affected in Siesta-KD mutants. These findings suggest that Siesta complexes control larval locomotion through a mechanism that is independent of H2AK118 ubiquitylation.

Many *Drosophila* regulators, including the Polycomb group proteins, are maternally supplied. In such cases, zygotic loss-of-function mutants derived from heterozygous parents begin development with maternally deposited wild-type mRNA and protein, which is often sufficient to support the early development and partially mask mutant phenotypes. To understand the role of maternally contributed Siesta, we used the strain where *sist^612^*/*sist^1012^* mutations were initially rescued by a transgenic *sist* copy, which was subsequently excised specifically in the germline using FLP recombinase (figs. S8 and S9). This approach generated Siesta-KO^mz^ mutants that lack both maternally deposited and zygotically expressed Siesta. The absence of Siesta protein and substantially reduced H2AK118ub in 0- to 24-hour-old embryos are evident from the Western blot analysis (Fig. 3D). None of the Siesta-KO^mz^ mutants progressed to the second instar larval stage, and the first instar larvae exhibited severely impaired locomotion compared to their zygotic counterparts (Fig. 3B and movies S1 and S2).

To assess whether the reduced locomotion was due to general sensory impairment, we tested the larvae’s response to hypoxia. In *Caenorhabditis elegans*, reduced oxygen levels trigger increased forward movement as an escape response (*61*, *62*). Similarly, both control and Siesta-KO^mz^ larvae increased their crawling speed when exposed to 1% oxygen, although the mutants remained substantially slower (fig. S10 and movies S3 and S4). These results suggest that the locomotion defect in *siesta* mutants is not caused by general sensory deprivation. The precise nature of the defect warrants further neuroanatomical investigation.

### *Drosophila* RYBP is not essential for viability and bulk H2AK118 ubiquitylation

In mammals, the presence of the RYBP subunit, or its paralog YAF2, distinguishes variant RING1/RING2 complexes from canonical PRC1, which instead incorporates one of the CBX subunits (*9*, *63*). The same applies to *Drosophila*, which encodes a single RYBP ortholog (*40*). The simplicity of the *Drosophila* system, with only one RYBP gene, provides an opportunity to dissect its functional contribution to RING1 complexes. Flies homozygous for *RYBP^KG08683^*, the only allele designated as loss-of-function in the literature, display reduced viability, substantial developmental delay and 90% female sterility (*64*). However, the *RYBP^KG08683^* allele is caused by a P-element insertion in the 5′UTR of *RYBP*, raising the possibility that it does not fully abolish gene function.

**Fig. 4.**
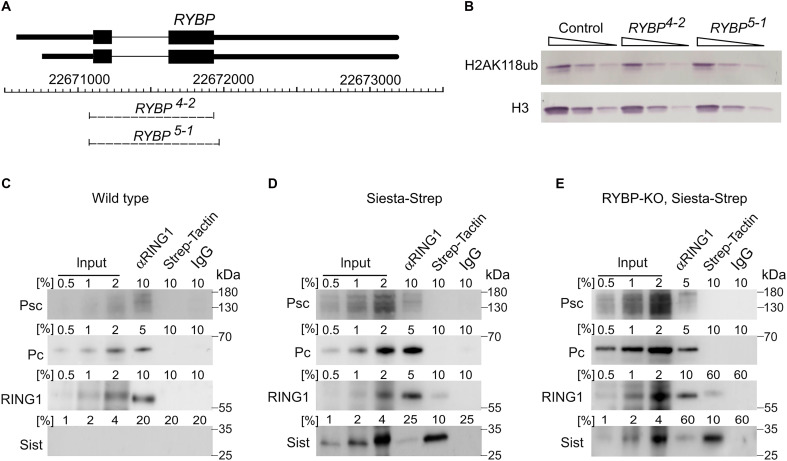
Drosophila RYBP is dispensable for the bulk of H2AK118 ubiquitylation. (**A**) Schematics of the *RYBP* locus. Two alternative transcripts above the coordinate scale (*dm6* genomic release) are shown with TSSs to the left. Thin lines indicate introns, and black boxes correspond to the coding parts. Dashed lines mark the extent of *RYBP^4-2^* and *RYBP^5-1^* deletions. (**B**) Threefold dilutions of the total protein extract from wild-type control, *RYBP^4-2^*, and *RYBP^5-1^* second instar larvae were analyzed by Western blot with antibodies against H2AK118ub and H3 (loading control). The assay shows no reduction of H2AK118ub in animals lacking RYBP. (**C** to **E**) Coimmunoprecipitation of Psc, Pc, RING1, and Sist from total nuclear protein extracts of wild type (C), Siesta-Strep (D), and RYBP-KO, Siesta-Strep embryos (E). The indicated fractions of material immunoprecipitated with anti-RING1, Strep-Tactin, and total rabbit IgG (negative control) were analyzed by Western blot with antibodies indicated to the left of each panel. Strep-Tactin–horseradish peroxidase (HRP) conjugate was used to detect the Twin-Strep–tagged transgenic Siesta protein. Positions of molecular weight markers (in kilodaltons) are shown to the right of each panel. Note that Siesta and RING1 coimmunoprecipitate even when RYBP is absent (E), although the signals are much weaker and require a larger fraction of precipitated material to be detected.

To address this uncertainty, we generated new *RYBP* mutations using CRISPR-Cas9–mediated genome editing. To this end, we designed guide RNAs (gRNAs) targeting the Cas9 endonuclease to the 5′UTR and 3′UTR of the *RYBP* transcript (Fig. 4A). As a result, we recovered two new alleles of the *RYBP* gene, which remove 880 bp (*RYBP^4-2^*) and 902 bp (*RYBP^5-1^*) and thus eliminate the entire ORF (Fig. 4A and fig. S11). To our surprise, and in contrast to Siesta-KO, these clean *RYBP* loss-of-function mutants (RYBP-KO) are homozygous viable, fertile, and can be maintained as stable stocks. This suggests that the developmental defects observed in *RYBP^KG08683^* flies are due to a second-site mutation or that the allele functions as a gain-of-function rather than a true null. Western blot analysis revealed no difference in bulk H2AK118ub levels between RYBP-KO and control flies (Fig. 4B), indicating that RYBP is not essential for global H2AK118 ubiquitylation in *Drosophila*.

To investigate the impact of RYBP loss on Siesta complexes, we generated the fly strain homozygous for RYBP-KO and Siesta-KO alleles complemented with a transgenic copy of *sist* expressing a fully functional Twin-Strep–tagged and Myc-tagged Siesta protein (*RYBP^4-2^*, *sist:Strep-Myc-sist*; *sist^1012^* referred to as RYBP-KO, Siesta-Strep). We then compared the coimmunoprecipitation of RING1 with Siesta, Psc, and Pc from nuclear extracts of RYBP-KO and Siesta-KO embryos and that from nuclear extracts of Siesta-KO embryos complemented with the same *sist* transgene as above (*sist:Strep-Myc-sist*; *sist^1012^*, referred to as Siesta-Strep). As illustrated in Fig. 4 (C to E), the coimmunoprecipitation of RING1 with Pc or Psc is not altered by the RYBP loss. In contrast, in the absence of RYBP, the coimmunoprecipitation of RING1 and Siesta is drastically (>6-fold) reduced, although it is still detectable (Fig. 4, C to E).

The Siesta-KO cells lose ~70% of the bulk H2AK118ub (*34*) (Fig. 1E), and it is well established that RING1 requires a PCGF partner for efficient ubiquitylation of H2AK118ub (*65*–*69*). Yet, we see no reduction in the bulk H2AK118ub levels in the RYBP-KO flies despite the substantial loss of coimmunoprecipitation between RING1 and Siesta when RYBP is absent. The latter suggests that the loss of RYBP does not affect the assembly of the Siesta-RING1 complex in vivo. We posit that, without RYBP, the complex becomes less stable and easily dissociates during protein extract preparation, resulting in substantially lower coimmunoprecipitation. The increased stability conferred by RYBP may explain the higher E3 ligase activity of RYBP-RING2 complexes toward H2AK119 in vitro (*9*, *27*).

### Epigenetic repression of developmental genes works without Siesta

**Fig. 5.**
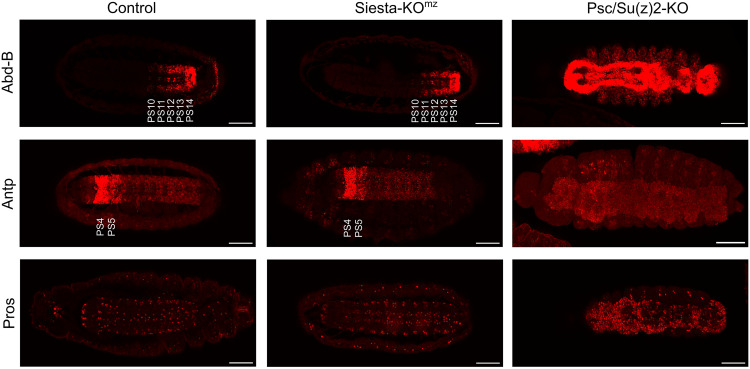
Siesta mutants maintain correct expression patterns of developmental genes. *Abd-B*, *Antp*, and *pros* expression was assayed by immunostaining of late-stage *Drosophila* embryos with the corresponding antibodies. The embryo genotypes [Control, Siesta-KO^mz^, and Psc/Su(z)2-KO] are indicated above the image panel. A single optical section is shown for representative embryos stained with anti–Abd-B antibodies. Maximum intensity projection (MIP) of five *Z* stacks is shown for embryos stained with anti-Antp antibodies, and the MIP of two *Z* stacks is shown for embryos stained with anti-Pros antibodies. PS10 to PS14 and PS4 and PS5 are indicated to highlight the *Abd-B* and *Antp* expression patterns in the control and Siesta-KO^mz^ embryos. Scale bars, 50 μm.

The Siesta-KO^mz^ larvae display no obvious homeotic transformations, suggesting that, in contrast to PRC1 subunits, Siesta is not essential for epigenetic repression of developmental genes. To further test this conjecture, we compared the expression of the developmental regulators *prospero* (*pros*), *Abdominal-B* (*Abd-B*), and *Antennapedia* (*Antp*) in Siesta-KO^mz^, Psc/Su(z)2-KO [animals homozygous for the *Su(z)2-1.b8* deletion produced by heterozygous parents] and control embryos. *Antp* and *Abd-B* are homeotic selector genes that encode transcription regulators necessary to specify the identity of embryonic parasegments 4 to 5 (PS4 and PS5) and PS10 to PS14, respectively. The *pros* gene is expressed in a set of cells in the central nervous system, specifying their fate. The correct spatial expression pattern of *Antp*, *Abd-B*, and *pros* is achieved by the competing action of transcriptional enhancers and epigenetic repression by the Polycomb system. As expected, embryos lacking a zygotic supply of the Psc and Su(z)2 proteins show wide misexpression of *Abd-B*, *Antp*, and *pros* in the nervous system (Fig. 5). In contrast, the spatial expression patterns of *Abd-B*, *Antp*, and *pros* in the Siesta-KO^mz^ do not differ from those in the control embryos (Fig. 5). These results indicate that Siesta complexes are not generally required for the epigenetic repression of *Drosophila* developmental genes.

### A high-throughput transgenic assay provides no evidence of transcriptional repression by H2AK118ub

Studies in mouse embryonic stem cells suggested that H2AK119 ubiquitylation (H2AK118ub in *Drosophila*) acts as a generic transcriptional repressor and a primary means by which the Polycomb system represses target genes (*70*). Contradicting this view, Pengelly and colleagues did not detect phenotypes characteristic of Polycomb group mutants in *Drosophila* deficient for H2AK118 ubiquitylation (*37*). Furthermore, transcriptome analyses of the mutants failed to detect any genes whose transcription significantly changed. The conventional RNA sequencing assay used in the study was designed to detect differences in the transcriptional output of a small subset of genes. Given the widespread distribution of H2AK118 ubiquitylation, its loss could have affected many genes at once, complicating the detection.

To overcome this limitation, we used the Thousand Reporters Integrated in Parallel (TRIP) assay (*71*), which enables genome-wide assessment of transcriptional activity by measuring how the same reporter gene behaves when integrated at different genomic locations. If H2AK118ub functions as a strong repressor, reporter constructs inserted into regions with high H2AK118ub—such as PRE-associated loci (Cluster I) or regions enriched in Siesta-dependent H2AK118ub (Cluster II)—should exhibit lower transcriptional output compared to insertions in regions with low H2AK118ub (Cluster III). The division of labor between PRC1 and Siesta-RING1 complexes provides for an additional test. If the model is correct, the transcription of reporter genes integrated into Cluster I should be substantially higher in Psc/Su(z)2-KO cells compared to that in the control cells. Conversely, reporters integrated in Cluster II regions should be transcribed more in Siesta-KO cells. Furthermore, insertions in both kinds of regions should produce more RNA in RING1-KO cells where H2AK118ub is globally depleted.

**Fig. 6.**
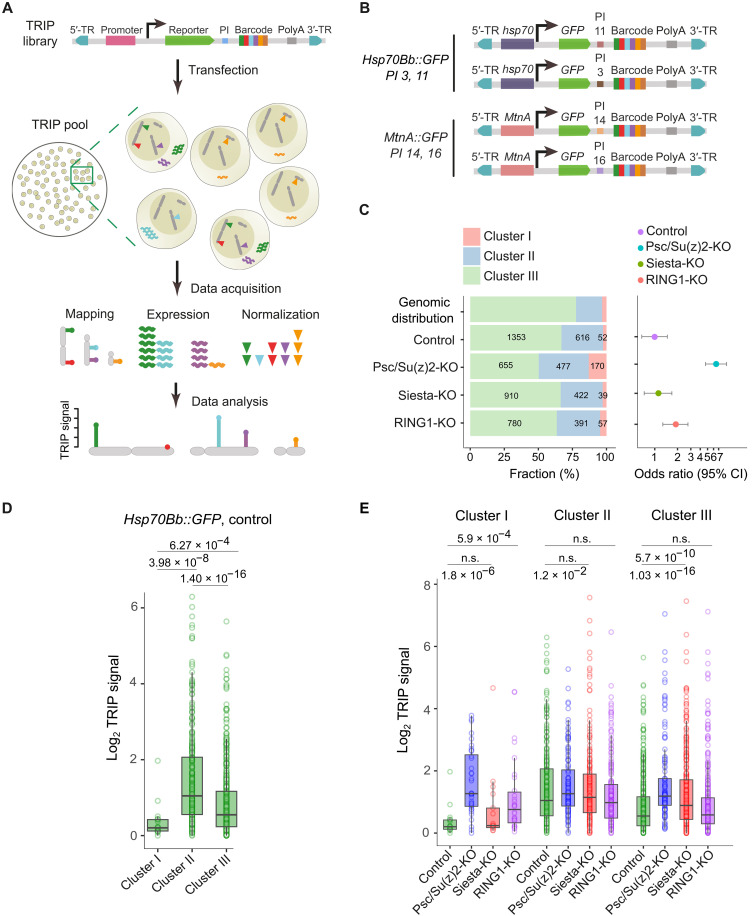
TRIP reveals no obvious repressive effect of H2AK118 ubiquitylation on transcription. (**A**) Schematic of the TRIP assay. The same color code is used to illustrate reporter constructs integrated at distinct genomic sites and the corresponding RNA products. (**B**) Schematic representation of the four TRIP libraries used for the assay. All constructs were integrated in the genome via piggyBac 5′ (5′-TR) and 3′ (3′-TR) terminal repeats, but each library was marked with a distinct promoter index (PI). (**C**) Stacked bar plots show fractions and numbers of transgenic insertions within Cluster I to III regions in different cell lines compared to the relative abundance of each region type within the *Drosophila* genome. The dot plot to the right indicates the odds ratios of integration within PRE-regulated genes compared to the rest of the genome within different cell lines. Whiskers mark the 95% confidence interval (CI). Note a significant increase in the odds of integrating into the PRE-regulated genes in the Psc/Su(z)2-KO cells. (**D**) TRIP signals for the *Hsp70Bb::GFP* transgenes integrated in three types of genomic regions in control cells. Here and in (E), the box plots indicate the median and span interquartile range with whiskers extending 1.5 times the range. The differences between the corresponding groups were tested for statistical significance using the Wilcoxon rank sum test, and *P* values are displayed above the box plots. (**E**) TRIP signals for the *Hsp70Bb::GFP* transgenes in different cell lines. n.s., not significant.

To test these predictions, we generated four TRIP libraries consisting of tens of thousands of reporter constructs flanked by inverted repeats from the *piggyBac* mobile element (Fig. 6A). All libraries contained an identical *GFP* reporter gene fused at the 3′ end to an 18-bp DNA segment with a random nucleotide sequence (barcode) (Fig. 6, A and B). In two of the libraries, the *GFP* reporter was placed under the control *Hsp70Bb* gene promoter, whereas in the two other libraries, the *GFP* transcription was controlled by the *Metallothionein A* (*MtnA*) gene promoter (*72*). Each library was distinguished by a specific 5-bp sequence (promoter index). The *Hsp70Bb* promoter can drive robust medium-level transcription of chromatin-integrated reporters due to its open chromatin architecture that favors RNA Pol II recruitment (*73*, *74*). The behavior of the *MtnA* promoter in reporters integrated into the genome is less studied, but the transcription from the *MtnA* promoter could be induced by supplementing the cell culture growth medium with copper ions (*72*).

An approximately equimolar mixture of all four TRIP libraries was transfected into control, Psc/Su(z)2-KO, Siesta-KO, and RING1-KO cells along with the plasmid expressing piggyBac transposase. The resulting pools of transgenic cells (TRIP cell pools) were used to map the genomic location of barcoded insertions by high-throughput sequencing of inverse PCR products. The transcriptional activity of the corresponding transgenes in each TRIP cell pool, grown on media with and without copper, was deduced by counting barcodes after sequencing RNA produced by transgenes. To account for variations in the growth rates of individual cells in a TRIP cell pool and potential biases during sequencing library preparations, the barcode counts were further normalized by counting the barcode sequences within genomic DNA isolated from the corresponding TRIP cell pools (Fig. 6A; for further details, see the Materials and Methods section). All assays were performed in duplicate for each TRIP cell pool, starting from independent cell cultures.

Across all promoter variants and indices, we analyzed 1228 to 2021 distinct reporter insertions per genetic background (Fig. 6C). In all cases, the largest fraction of the insertions occurred within regions with low H2A118ub (Cluster III regions), followed by regions with high H2A118ub produced by Siesta-RING1 complexes (Cluster II regions) and a smaller number of insertions into the PRE-equipped loci (Cluster I regions). This distribution largely reflects the relative genomic representation of Clusters I to III (Fig. 6C). We noted sevenfold higher odds of insertions into PRE-equipped loci (Cluster I regions) in Psc/Su(z)2-KO cells compared to control, Siesta-KO, and even RING1-KO cells. This suggests that the PRE-equipped loci hinder the integration of *piggyBac*-based transgenes. This hindrance is dependent on Psc and/or Su(z)2 but not on H2AK118 ubiquitylation.

Analysis of TRIP signals from *Hsp70Bb::GFP* transgenes integrated into the genome of control cells shows that the transgenes integrated into PRE-equipped loci (Cluster I regions) are transcribed less than those integrated into Cluster II and Cluster III regions (Fig. 6D). This is expected and agrees with previous observations that P-element–based transgenes become repressed by the Polycomb system when integrated close to PREs (*48*, *75*). These results confirm that our TRIP assay is sufficiently sensitive to detect the repressive effect of the Polycomb system. Unexpectedly, and contrary to the model in which H2AK118ub acts as a strong transcriptional repressor, the transgenes integrated into regions with high H2AK118ub (Cluster II; Figs. 2C and 6D) are more transcriptionally active compared to transgenes integrated into regions with lower H2AK118ub (Cluster III; Figs. 2C and 6D).

Consistent with the observations above, the transgenes integrated into PRE-equipped loci (Cluster I regions) show significantly higher transcriptional activity in Psc/Su(z)2-KO and RING1-KO cells compared to that in the control and Siesta-KO cells (Fig. 6E). This reinforces the argument that transgenes integrated in these regions are repressed by the Polycomb system and that our TRIP assay is sufficiently sensitive to detect the repression. Contrary to the model proposing H2AK118ub as a transcriptional repressor, transgenes integrated in Cluster II and III regions are transcribed at the same level in RING1-KO cells, completely devoid of this modification (Fig. 6E). In line with our previous findings that ablation of Psc and Su(z)2 leads to a weak but significant increase in transcription everywhere in the genome (*35*), *Hsp70Bb::GFP* transgenes integrated into Cluster II and III regions display higher transcription in Psc/Su(z)2-KO cells (Fig. 6E). This effect is more pronounced for the generally lower-expressing insertions in Cluster III regions and does not correlate with the extent of H2AK118ub loss. The latter is significantly greater in Siesta-KO cells, in which transcription is essentially the same for insertions in Cluster II regions and just slightly elevated for insertions in Cluster III regions compared to control cells (Fig. 6E).

The examination of TRIP signals from *MtnA::GFP* transgenes leads to similar conclusions. These transgenes display generally low transcription when cells are grown in the media without Cu^2+^ ions (fig. S12A). Nevertheless, the transcription of transgenes integrated into PRE-equipped loci (Cluster I regions) is weaker compared to those integrated elsewhere (fig. S12A). The TRIP signals from our *MtnA::GFP* transgenes increase when cells are grown on media supplemented with copper (fig. S12, B and C). The median increase is lower than anticipated from the original study with transiently transfected constructs (*72*), suggesting that the *MtnA* promoter is far less efficient in inducing transcription of chromatin-integrated transgenes. Even in the presence of Cu^2+^ ions, the transgenes integrated into PRE-equipped loci (Cluster I regions) are transcribed significantly less compared to those integrated elsewhere. Yet, we see no difference between *MtnA::GFP* transgenes integrated into Cluster II and Cluster III regions despite the former displaying significantly higher H2AK118ub ChIP-seq signals (fig. S12C). Like *Hsp70Bb::GFP* transgenes, the *MtnA::GFP* transgenes integrated into PRE-equipped loci (Cluster I regions) show significantly higher transcriptional activity in Psc/Su(z)2-KO and RING1-KO cells compared to that in the control and Siesta-KO cells (fig. S12D). On the other hand, the effects of Psc/Su(z)2, Siesta, and RING1 knockout on the transcription of transgenes integrated away from PREs are weak and variable. We see no correlation between the extent of transcription and the loss of H2AK118ub.

To summarize, our TRIP assay demonstrates that, regardless of the promoter, transgenes integrated in the PRE vicinity tend to transcribe less than transgenes integrated elsewhere. The apparent transcriptional repression, which we attribute to the action of the Polycomb system, is relieved by removal of Psc/Su(z)2 or RING1 but not Siesta. However, the transgenes not influenced by the Polycomb system (i.e., integrated away from PREs) display no correlation between transcriptional activity and the degree of H2AK118ub within its integration site. Likewise, they show no consistent transcriptional changes correlated to the loss of H2AK118ub in various mutant backgrounds. We conclude that the repressive effect of H2AK118 ubiquitylation, if any, is much weaker compared to transcriptional repression by the protein complexes bound at PREs.

## DISCUSSION

Epigenetic repression of developmental genes requires canonical PRC1, which acts by interacting with H3K27me3 to propagate the repressed state of regulated genes. Canonical PRC1 is also an E3 ligase that can deposit a single ubiquitin molecule on K118/119 of histone H2A. This enzymatic activity is shared with other complexes formed by the RING1/RING2 subunit of canonical PRC1, although the latter cannot bind H3K27me3. It has been proposed that H2A ubiquitylation is an essential part of the repressive mechanism and that variant RING1/RING2 complexes evolved from ancestral canonical PRC1 to diversify the Polycomb system and enable the evolution of vertebrate-specific traits (*70*). Yet, tracing of genes encoding core components of RING1/RING2 complexes in genomes from diverse animal clades argues that variant RING1/RING2 complexes emerged early in animal evolution (*12*) and therefore neither all variant complexes nor H2AK118/119ub should be automatically assumed to act as epigenetic transcriptional repressors. Our findings strongly support this view. In *Drosophila*, we show that Psc/Su(z)2-RING1 and Siesta-RING1 complexes monoubiquitylate H2A across distinct genomic regions. Furthermore, the Siesta protein, is dispensable for epigenetic repression of homeotic genes and controls larval locomotion independently of H2A ubiquitylation. Exploiting the division of labor between Psc/Su(z)2-RING1 and Siesta-RING1 complexes, we used the TRIP assay to demonstrate that H2A ubiquitylation has no major repressive effect on transcription. Below, we discuss the implications of our findings in more detail.

In the current literature, RING1/RING2 complexes are customarily referred to as variants of PRC1, providing a convenient nomenclature (e.g., PRC1.1, PRC1.2, etc.). However, this terminology implies common function of all RING1/RING2 complexes in epigenetic repression and may be misleading. For example, a recent study considered RING1/RING2 complexes as a functionally coherent group and reported no coupling in the evolution of the PRC1 and PRC2 parts of the Polycomb system (*13*). This inference seems premature until the coevolution of canonical PRC1 and PRC2 is examined independently of other RING1/RING2 complexes. To prevent possible confusion in the future, we suggest revising the current nomenclature and reserving the Polycomb Repressive Complex 1 name for canonical PRC1.

Consistent with earlier observations (*21*, *35*), we find that most of the nonrepeated *Drosophila* genome is packaged into nucleosomes monoubiquitylated at H2AK118. Notably, the bulk of this widespread H2AK118 ubiquitylation is achieved without stable binding of RING1 complexes. PREs seem to be the only sites in the *Drosophila* genome stably bound by RING1 complexes. These complexes, likely canonical PRC1, contain Psc or Su(z)2 proteins and produce most of the H2AK118ub at PRE-regulated genes. How PREs tether PRC1 is not well understood. According to one model, PREs are composed of binding sites for multiple DNA binding proteins that combine individually weak interactions with PRC1 to yield the robust tethering (*76*). Which of the PRC1 subunit(s) interact with these DNA binding proteins is not known, but our observations suggest it is not RING1. Otherwise, Siesta-RING1 complexes would also be bound to PREs.

Changes of RING1 levels in Siesta-KO and Psc/Su(z)2-KO cells indicate that most of the *Drosophila* RING1 protein resides in the complex with Psc/Su(z)2. Yet, the hit-and-run action of less abundant Siesta-RING1 complexes produces most of the steady-state H2AK118ub. This suggests that Siesta-RING1 complexes are more active E3 ligases. Stimulation by the RYBP subunit (*9*, *27*) or the differences in the biochemical properties of distinct PCGF proteins (*28*) were proposed as explanations of the increased activity of variant RING1/2 complexes. Our genetic data support the latter view. Flies lacking the RYBP function are viable and fertile and show no detectable differences in the bulk H2AK118ub, although the RYBP loss results in substantially lower recovery of Siesta-RING1 complexes by coimmunoprecipitation. This suggests that RYBP is dispensable for the assembly and activity of the Siesta-RING1 complexes in vivo but makes the complexes more resistant to dissociation during protein extract preparation. The increased stability may explain why RYBP-RNF2 complexes perform better in ubiquitylating H2AK119 in vitro (*9*, *27*).

In *Drosophila* cells, RING1 protein appears to be limiting and unstable when not incorporated into a complex. This makes distinct PCGF proteins compete for the common RING1 partner. Because PRC1 and Siesta-RING1 complexes have distinct functions, it is important to maintain the relative abundance of the two kinds of complexes in the cell. This balance appears to be regulated, at least in part, through the expression of *Psc* and *Su(z)2*. These two genes are located next to each other in a locus that includes multiple PREs bound by PRC1 and PRC2 (*6*, *48*). This configuration generates a feedback loop where a reduction in Psc or Su(z)2 increases the transcription of the *Psc* and *Su(z)2* genes (*49*), tilting the balance toward PRC1 assembly. On the other hand, when Siesta levels decline, more RING1 protein is available for incorporation into PRC1, increasing its intranuclear concentration and enhancing transcriptional repression of *Psc* and *Su(z)2* genes. This feedback loop may be evolutionarily conserved. In humans, the *BMI1* gene, encoding one of the two PCGF proteins specific to canonical PRC1 and an ortholog of *Psc/Su(z)2*, is also regulated by the Polycomb system (*77*). Whether the second human ortholog, *MEL18*, is similarly regulated by the Polycomb system and whether loss of PCGF proteins specific to the variant RING1/RING2 complexes reduces transcription of the *BMI1* or *MEL18* genes are fascinating open questions to address in the future.

The locomotion defect of the Siesta-KO mutants parallels behavioral abnormalities caused by mutations of the mammalian *Autism Susceptibility Gene 2* (*AUTS2*) (*24*) and locomotion defects of the *Drosophila Tay bridge* (*Tay*) (*78*) genes encoding the corresponding subunits of one of the Siesta-RING1 complexes (*9*, *40*). Consistent with our findings, mammalian AUTS2-PCGF3/5-RING1/2 complexes (analogs of the Tay-Siesta-RING1 complex) act independently of H2AK119 ubiquitylation and appear to stimulate transcription of a subset of genes (*23*, *24*). Although we have clear evidence that Siesta complexes (likely the Tay-Siesta-RING1) control larval locomotion independently of H2AK118 ubiquitylation, it is premature to conclude that their action does not require ubiquitin E3 ligase activity. It is possible that, in addition to H2AK118, the Tay-Siesta-RING1 complexes ubiquitylate yet unknown nonhistone protein(s). These proteins may represent the biologically relevant target of Siesta-RING1 ubiquitylation, whereas H2AK118ub may be an abundant side product. It is worth pointing out that the truncated RING1-I48A protein (or the analogous truncated version of mammalian orthologs), used to benchmark the effects of E3 inactivation of RING1 complexes, shows drastically impaired E3 ligase activity toward H2AK118. However, this may not hold true for the hypothetical nonhistone substrate. The same line of reasoning applies to the E3 ubiquitin ligase activity of canonical PRC1. The findings and mutants presented in our work provide an important framework to test these hypotheses and search for the nonhistone targets of RING1 ubiquitylation.

## MATERIALS AND METHODS

### Generation of *RYBP* mutant alleles

The two gRNAs were synthesized with the GeneArt Precision gRNA Synthesis Kit (Invitrogen). The following oligonucleotides were used to amplify the DNA templates for in vitro transcription: gRNA1-fw (5′-TAATACGACTCACTATAGGGATTGCGGTTACTCCA-3′), gRNA1-rev (5′-TTCTAGCTCTAAAACCTGGAGTAACCGCAATCC-3′), gRNA2-fw (5′-TAATACGACTCACTATAGGACAGCCGGAGTTAGAG-3′), and gRNA2-rev (5′-TTCTAGCTCTAAAACCCTCTAACTCCGGCTGTC-3′). To prepare gRNA-Cas9 ribonucleoproteins (RNPs), a mixture of TrueCut Cas9 (1 μg/μl; Invitrogen) with 0.25 ng/μl of each gRNA was incubated at room temperature, and the solution was cleared by centrifugation. The resulting supernatant was used for injection of *w^1118^* embryos [Bloomington Drosophila Stock Center (BDSC) strain #5905]. Individual F0 males were crossed with isogenic balancer stock *w^1118^; sna^Sco^/SM6a* females (BDSC strain #5907). Their male progeny was again individually crossed to *w^1118^; sna^Sco^/SM6a* females, and after 7 days of breeding, males were euthanized for a PCR deletion screen using primers 5′-TCGAAGTAGGGTTCTCTGCC-3′ and 5′-TGAGTGCGGCTTTAATTGCGTT-3′. The homozygous mutant strains were further analyzed by Sanger sequencing.

### Survival assay

To assess the viability and determine the lethal stage, first instar larvae of a genotype of interest were individually picked on the basis of the green fluorescent protein (GFP) expression and put into apple juice agar plates supplemented with a small block of regular fly media. After 36 to 40 hours, second instar larvae were transferred to vials containing fly media (50 animals per vial) and their development followed by recording the number of prepupae, pupae, and hatched adults.

### Generation of Twin-Strep-Myc-Siesta expressing transgenes

To generate a transgenic construct expressing tagged Siesta protein under control of the endogenous *Sist* promoter, the 5′ and 3′ parts of the *Sist* locus (3L:16,587,861-16,590,261, *dm6* genomic coordinates) were PCR amplified separately using genomic DNA as a template and the L(3)73 genomic-For (5′-CGGGCCCCCCCTCGACCACTATGCACATAGACAC-3′) and iPCR-L(3)73-Rev (5′-CATGCTGGCCCCCCGATTG-3′) primers for the 5′ part and L(3)73 Ah genomic-Rev (5′-CGGTGGCGGCCTCGACAAAGGCTACCACGTCCT-3′) and iPCR-L(3)73-For (5′-GAGCGGCGCGTGAAGCTGAAG-3′) for the 3′ part, respectively. The resulting fragments were combined to flank the synthetic DNA sequence encoding tandem Twin-Strep and Myc tags (amino acid sequence ASWSHPQFEKGGGSGGGSGGGSWSHPQFEQKLISEEDLEERRVK) and introduced into the pW-attB vector (*79*) by the In-Fusion reaction, yielding the Endo-L(3)73 Ah–pW-attB construct. The construct was injected into *y^1^, M{vas-int.Dm}ZH-2A, w^−^; M{3×P3-RFP.attP}ZH-51D* embryos by BestGene.

### Generation of maternal, zygotic *siesta* mutants

The Siesta-KO^mz^ mutants were generated as described in (*80*). To this end, the pUMR-FLAP construct was digested with Eco RI and combined with the DNA fragment produced by PCR using l(3)_FLAP_For (5′-TACGAGATCTGAATTCCACTATGCACATAGACACCATA-3′) and l(3)_FLAP_Rev (5′-GAACTTCGAAGAATTCAAAGGCTACCACGTCCTC-3′) primers and the DNA of Endo–L(3)73 Ah–pW-attB as a template using the In-Fusion reaction. The resulting construct was injected into *y^1^, M{vas-int.Dm}ZH-2A, w^−^; M{3×P3-RFP.attP}ZH-51D* embryos by BestGene.

To generate *w^−^/w^−^; +/+; sist^1012^, {UASp-FLPo, w^+^}VK33, y^+^/TM3, Ser, {Act::GFP, w^+^} e* females; *+/+; sist^1012^/ {UASp-FLPo, w^+^}VK33, y^+^* females were crossed to *+/+; Su(z)12^4^, P{w^+^mC UAS-RAS^V12^}/TM3, Sb, e* males. *+/+; sist^1012^, {UASp-FLPo, w^+^}VK33, y^+^/ TM3, Sb, e* males were then crossed to *sist^1012^/TM3, Ser, {Act::GFP, w^+^}, e* females to generate *+/+; sist^1012^, {UASp-FLPo, w^+^}VK33, y^+^/ TM3, Ser, {Act::GFP, w^+^}, e* flies.

To generate *P{<sist::siesta, UAS::GFP<}ZH-51D; P{w^+mC^ = GAL4::VP16-nos.UTR}CG6325^MVD1^, sist^612^* males; *+/+; sist^612^/TM3, Ser, {Act::GFP, w^+^}, e* females were crossed to *If/ CyO ; P{w^+mC^ = GAL4::VP16-nos.UTR}CG6325^MVD1^/ TM3, Sb, e* males. Then, *+/ CyO; sist^612^/ P{w^+mC^ = GAL4::VP16-nos.UTR}CG6325^MVD1^* females were crossed to *+/+; Su(z)12^4^, P{w^+^mC UAS-RAS^V12^}/TM3, Sb, e* males. The resulting *+/ CyO; sist^612^, P{w^+mC^ = GAL4::VP16-nos.UTR}CG6325^MVD1^/ TM3, Sb, e* males were crossed to *+/+;sist^1012^/TM3, Ser, {Act::GFP, w^+^}, e* females to generate *+/ CyO; sist^612^, P{w^+mC^ = GAL4::VP16-nos.UTR}CG6325^MVD1^*/ *TM3, Ser, {Act::GFP, w^+^}, e* females. These were then crossed to *If/ CyO ; TM6, Tb/MKRS, Sb* to generate *+/CyO; sist^612^, P{w^+mC^ = GAL4::VP16-nos.UTR}CG6325^MVD1^/ MKRS, Sb* females. Later, they were crossed to *P{<sist::siesta, UAS::GFP<}ZH-51D/If;+/ TM3, Ser, {Act::GFP, w^+^}, e* males to generate *P{<sist::siesta, UAS::GFP<}ZH-51D/CyO; sist^612^, P{w^+mC^ = GAL4::VP16-nos.UTR}CG6325^MVD1^/ TM3, Ser, {Act::GFP, w^+^}, e.* These were then backcrossed to each other to generate the final *P{<sist::siesta, UAS::GFP<}ZH-51D; P{w^+mC^ = GAL4::VP16-nos.UTR}CG6325^MVD1^, sist^612^* flies.

To generate the Siesta-KO^mz^ embryos, the *P{<sist::siesta, UAS::GFP<}ZH-51D; P{w^+mC^ = GAL4::VP16-nos.UTR}CG6325^MVD1^, sist^612^* males were crossed to the *w^−^/w^−^; +/+; sist^1012^, {UASp-FLPo, w^+^}VK33, y^+^/TM3, Ser, {Act::GFP, w^+^}, e* females. From the resulting F1 progeny, males and females of the *P{<sist::siesta, UAS::GFP<}ZH-51D/+; P{w^+mC^ = GAL4::VP16-nos.UTR}CG6325^MVD1^, sist^612^/sist^1012^, {UASp-FLPo, w^+^}VK33, y^+^* genotype were crossed with each other, which resulted in embryos that lack maternal and zygotic Siesta protein. Control embryos were generated by crossing Oregon R females with *P{<sist::siesta, UAS::GFP<}ZH-51D; P{w^+mC^ = GAL4::VP16-nos.UTR}CG6325^MVD1^, sist^612^* males. The F1 progeny were then crossed with each other. Because there was no FLP recombinase source, the transgenic cassette was not excised (fig. S8).

### Locomotion assay

The locomotion of *Drosophila* second instar larvae was recorded and analyzed as previously described (*81*). Briefly, second instar larvae were transferred to apple juice agar plates supplemented with 0.1% bromophenol blue and allowed to acclimate for 30 to 60 s. A mobile phone with a 48-megapixel camera (recording resolution of 480 by 640 pixels) was used to capture videos of moving larvae. Four to six larvae were tracked in every video for a minimum of 90 s in a thermally controlled room set to 25°C. The WrMTrck ImageJ plugin (*82*) was used to analyze the videos. For each larva, the trimmed mean motion speed was calculated by omitting video frames with 10% of the highest and 10% of the lowest speeds to reduce background noise. The locomotion of first instar larvae was assayed as follows. The first instar larvae were placed on apple juice agar plates and allowed to acclimate for 30 to 60 s. Videos were acquired using a FLIR camera (recording resolution of 480 x 640 pixels) mounted on a Zeiss Stemi-508 microscope as previously described (*83*) and analyzed using WrMTrck ImageJ plugin as described above except that all video frames were analyzed and mean motion speed was calculated. To examine the hypoxia-evoked locomotory response, the larvae were sealed in a microfluidic chamber, and defined gases were delivered as previously described (*83*).

### Immunostaining and microscopy

*Drosophila* embryos were fixed and stained as previously described (*84*) with the following modifications. The embryos were dechorionated for 3 min in 7% sodium hypochlorite, and the blocking solution contained 5% newborn calf serum in Phosphate-Buffered Saline with Tween (PBST) (137 mM NaCl, 2.7 mM KCl, 10 mM Na_2_HPO_4_, 2 mM KH_2_PO_4_, and 0.1% Tween 20) instead of normal goat serum. The stained embryos were mounted on a glass slide and imaged with a Leica SP8 confocal microscope. Immunostaining and imaging of polytene chromosomes were done as described in (*85*). The antibodies used are listed in table S2.

### Generation of Siesta-KO, RING1-KO, and control cell lines

The Siesta-KO cell lines were derived from homozygous *sist^612^* embryos using the *Ras*^*V12*^-mediated transformation approach by Simcox and colleagues (*47*) with modifications described in (*34*). The *Ras*^*V12*^-transformed but otherwise wild-type control cell line (Ras17) was derived in parallel using the same procedure.

Cultured cell lines homozygous for the deletion of the *Sce* gene (RING1-KO cells) were derived by CRISPR-Cas9–mediated genome editing of Ras17 cells. Generation of *Drosophila* knockout cell lines using CRISPR-Cas9–mediated genome editing has two challenges. First, cultured *Drosophila* cells are difficult to clone due to poor cell proliferation in dilute cultures. Second, the frequency of generating deletions in both copies of a gene is low. This is likely due to the strong somatic pairing of homologous chromosomes, which causes most of the double-stranded DNA (dsDNA) breaks induced by Cas9 to be repaired by homology-directed DNA repair using the unedited homolog as a template. To overcome these limitations, we developed a new cell cloning protocol and a two-step CRISPR-Cas9 editing strategy, which is described below.

To isolate single-cell clones, cultured *Drosophila* cells are diluted 100, 1000, and 10,000 times and plated in a 6-well plate covered with 0.1% gelatine. After 5 to 7 days, clusters of cells originated from mitotic divisions of individual cells are manually picked under a dissection microscope and reseeded in a 96-well plate. In 2 to 3 weeks, successfully proliferating clonal cultures are reseeded into a 24-well plate and genotyped. For the two-step CRISPR-Cas9 editing, four single guide RNAs (sgRNAs) are designed to generate a deletion of interest (fig. S13). In the first editing round, the cells are transfected with the Cas9/gRNA complex that includes an outer pair of gRNAs (fig. S13) and the resulting cell population is used to derive single-cell clonal cultures. At this stage, all of the resulting cultures are heterozygous with only one allele bearing the desired deletion. In the second step, two independently isolated heterozygous clones are treated with the inner pair of sgRNAs. At this step, the wild-type template for homology-directed DNA repair is not available, so in most cases, the deletion allele obtained in the first step is used as a template instead. The resulting cell population is used to derive single-cell clonal cultures. After the second round of editing and cloning, more than half of the derived cultured cell lines are homozygous for the desired deletion.

To generate cultured cell lines homozygous for the deletion of the *Sce* gene (RING1-KO cells), Ras17 cells were subjected to two rounds of CRISPR-Cas9–mediated genome editing as outlined above. For each editing round, 50 pmol of each sgRNA (Synthego) was mixed with 60 pmol of Cas9 nuclease (IDT) and delivered to 1 million cells by electroporation with the Neon Transfection System 10 μl kit from Thermo Fisher Scientific with a 1800-V pulse for 10 ms twice according to the manufacturer’s instructions. In the first editing round, the Cas9 complexes with gRNA-dSce-ex1.1 (5′-UGGUGUGAAAAUGACGUCGC-3′) and gRNA-dSce-ex2.1 (5′-CUAUGGAAAUGUAUUACUCG-3′) were used for treatment, which resulted in the *Sce* deletion from one of the homologous chromosomes, detected in several clonal cell cultures (fig. S14). Two such cultures (cell lines Sce-KO63-2 and Sce-KO66) were used for the second editing round using Cas9 in the complex with gRNA-dSce-ex1.2 (5′-CCCGGCGCCAAACAAAACGU-3′) and gRNA-dSce-ex2.2 (5′-AAAGCAUACAGACAUUAGAA-3′). The resulting cell population was used to derive single-cell clonal cultures, some of which were genotyped by PCR amplification of the junction and sequencing the PCR fragments (fig. S14). Half of the genotyped clonal cultures were homozygous for the deletion allele obtained in the first editing round due to homology-directed repair of the DNA breaks produced during the second editing round. The other half of the clones harbored two different alleles: one allele with the original deletion from the first editing round and the second allele originated from nonhomologous end joining of dsDNA breaks produced during the second editing round. The nucleotide sequences of PCR primers are listed in table S3. All cell lines were cultured at 25°C in Schneider’s medium (BioConcept) supplemented with 10% of heat-inactivated fetal bovine serum (Sigma-Aldrich), streptomycin (0.1 mg/ml), and penicillin (100 U/ml) (Gibco) under sterile conditions.

### Protein coimmunoprecipitation

#### Embryo collection

Embryos were collected twice daily from 20 ventilated 250-ml Erlenmeyer flasks assembled with apple juice plates with a knob of yeast paste and populated with 300 to 400 3- to 5-day-old flies. The harvested embryos were placed in a 70-μm Corning cell strainer, washed with running deionized water, rinsed with embryo wash solution (0.04% Triton X-100 and 120 mM NaCl) and again with running deionized water. Then, embryos were partially dried and packed into a “tablet” by blotting the mesh with paper tissues, weighed, wrapped in aluminum foil, and flash frozen in liquid nitrogen. Frozen embryos were stored at −80°C until protein extraction.

#### Nuclear protein extraction

Approximately 12 g of embryos was placed in a DIY nylon mesh-bottom basket and submerged in a glass with 3.5% bleach for 4 min, while breaking lumps and gently stirring with a plastic spoon, to remove the chorions. The dechorionated embryos were washed extensively with deionized water and transferred into a prechilled 40-ml Wheaton glass homogenizer. Embryos were resuspended in 10 ml of embryo buffer [15 mM Hepes (pH 7.6), 10 mM KCl, 5 mM MgCl_2_, 0.1 mM EDTA, 0.5 mM EGTA, 350 mM sucrose, 1 mM dithiothreitol (DTT), 0.5 mM phenylmethylsulfonyl fluoride (PMSF), and 1x protease inhibitor cocktail cOmplete, EDTA-free (Roche)] and homogenized with 10 strokes of pestle A, followed by 20 strokes of pestle B. The resulting homogenate was filtered through a 70-μm cell strainer, and the debris was resuspended in 10 ml of embryo buffer and given an additional 10 strokes with pestle B, followed by filtration. The combined homogenate was centrifuged for 15 min at 10,000*g* and 4°C. The supernatant was carefully aspirated and discarded, and fat deposits were wiped from the walls with tissue paper. Nuclei were resuspended in 10 ml of embryo buffer, trying not to disturb the yellow egg yolk layer at the bottom, and the centrifugation was repeated. The resulting nuclei were resuspended in 2 ml of low-salt nuclear buffer [15 mM Hepes (pH 7.6), 20% glycerol, 1.5 mM MgCl_2_, 20 mM KCl, 0.1 mM EDTA, 1 mM DTT, 0.5 mM PMSF, and complete protease inhibitor cocktail, EDTA-free (Roche)], and the concentration of KCl in the suspension was adjusted to 400 mM with 3 M KCl solution. The suspension was incubated on ice with occasional gentle stirring (every 5 to 10 min) and then another 30 min inside a prechilled ultracentrifuge (meanwhile, a vacuum was reached). The suspension was centrifuged for 1 hour at 4°C and 100,000*g* to separate into four layers. Of those, the dense top fat layer was mechanically removed with a 5-μl microbiological loop, the transparent off-white nuclear extract (about 2.2 to 2.5 ml total) was split into 500-μl aliquots, flash frozen in liquid nitrogen, and stored at −80°C, whereas the opaque grayish liquid phase and solid bottom phase were discarded.

#### Antibody cross-linking to magnetic beads

The cross-linking was performed according to the manufacturer’s instructions with the following modifications. Dynabeads (Thermo Fisher Scientific) were separated from the storage solution by incubation on a magnetic stand and washed two times in 200 μl of wash buffer [1x phosphate-buffered saline (PBS), 0.02% NP-40, 1 mM DTT, 0.5 mM PMSF, and complete protease inhibitor cocktail, EDTA-free (Roche)] and resuspended in 200 μl of wash buffer. One microgram of the anti-RING1 antibody or 1 μg of normal rabbit immunoglobulin G (IgG) (Sigma-Aldrich, NI01-100UG) was added to the bead suspension and incubated for 30 min at room temperature while slowly rotating. Antibody-bound Dynabeads were washed one time with wash buffer, two times with 200 μl of conjugation buffer [20 mM sodium phosphate and 0.15 M NaCl (pH 8)] and resuspended in 125 μl of conjugation buffer. The cross-linking was initiated by adding 125 μl of 10 mM BS3 [bis(sulfosuccinimidyl)suberate] solution (Thermo Fisher Scientific), followed by slow rotation at room temperature for 30 min. The reaction was stopped by quenching with 12.5 μl of 1 M Tris (pH 7.5) for 15 min with slow rotation. The resulting affinity resins were washed three times with low-salt nuclear buffer supplemented with 0.02% NP-40 and 1 mM DTT and stored at 4°C.

#### Immunoprecipitation

Nuclear extracts were defrosted on ice and dialyzed two times for 2 to 3 hours against 14 ml of 25 mM Hepes-KOH (pH 7.6), 0.1 M KCl, 1.5 mM MgCl_2_, 0.1 mM EDTA, 20% (v/v) glycerol, 1 mM DTT, 0.5 mM PMSF, and cOmplete protease inhibitor cocktail, EDTA-free (Roche) using Slide-A-Lyzer MINI Dialysis Devices, 10K MWCO (Thermo Fisher Scientific) placed on ice with slow rocking. The affinity resins were washed two times with low-salt nuclear buffer supplemented with 0.02% NP-40 and 1 mM DTT and combined with 100 μl of the dialyzed extracts precleared by centrifugation. The resulting suspensions were adjusted to contain 0.02% of NP-40 and incubated overnight with slow rotation at 4°C. The resins were washed three times with 500 μl of wash buffer and once with wash buffer diluted 1:10 with water. For washing, Dynabeads were gently resuspended in the buffer by pipetting and separated on a magnetic stand, whereas Strep-Tactin Sepharose (IBA) beads were rotated for 5 min before centrifugation (2 min, 500*g*). The protein bound to antibody-coupled Dynabeads was eluted with 100 μl of 1x NuPAGE LDS sample buffer (Invitrogen) supplemented with 5% β-mercaptoethanol at 98°C for 10 min. The protein bound to Strep-Tactin Sepharose was eluted by 10-min incubation with 40 μl of 2x NuPAGE LDS sample buffer followed by incubation with an additional 50 μl of 1x NuPAGE LDS sample buffer and combining the eluates. To prepare the matching immunoprecipitation input samples, the dialyzed nuclear extracts were combined with 4 volumes of acetone (prechilled to −20°C) and subjected to protein precipitation overnight at −20°C. The precipitated protein was pelleted by centrifugation, washed with 50% acetone, and air-dried for 10 min. Protein pellets were resuspended in 1x NuPAGE LDS sample buffer (200 μl per 1 input volume) for 10 min at 98°C, homogenized with a pestle, vortexed, heated for an additional 10 min, and centrifuged to remove the remaining insoluble material.

### Reverse transcription quantitative PCR

Total RNA from 5 × 10^6^ cells was isolated using TRIzol (Invitrogen), and 2 μg was used for random primed synthesis of the cDNA with First Strand Synthesis Kit (Thermo Fisher Scientific). The control reaction, omitting reverse transcriptase, was always run in parallel. cDNA was treated with RNase A to remove the RNA template, purified with the Zymo PCR purification kit, and eluted with 200 μl of elution buffer. The amount of cDNA for a gene of interest in the given preparation was analyzed by qPCR and was expressed as a fraction of *RpL32* cDNA. Serial dilutions of genomic DNA were used to make the standard curve. qPCR analysis was performed using a Bio-Rad CFX Connect Real Time PCR instrument in a total reaction volume of 10 μl containing 4 μl of cDNA solution, 1x qPCRBIO SYGreen Master Mix (PCRBioSystems), and the corresponding primers (200 nM) (sequences indicated in table S3).

### Chromatin immunoprecipitation

#### Preparation of MNase-digested chromatin from cross-linked Drosophila cultured cells

Cells were cross-linked by adding the formaldehyde solution directly to the cell culture to a final concentration of 1.8% and incubating for 10 min at 25°C. The reaction was stopped by adding glycine (pH 7.0) to a final concentration of 0.125 M. Cross-linked cells were washed once in cold ChIP wash buffer A [10 mM Hepes (pH 7.0), 10 mM EDTA (pH 8.0), 0.5 mM EGTA (pH 8.0), and 0.25% Triton X-100] for 10 min at 4°C and once in cold ChIP wash buffer B [10 mM Hepes (pH 7.6), 100 mM NaCl, 1 mM EDTA (pH 8.0), 0.5 mM EGTA (pH 8.0), and 0.01% Triton X-100] for 10 min at 4°C. Approximately 0.5 ml of pelleted cross-linked cells was resuspended in 5 ml of cold TE buffer [10 mM tris-HCl (pH 8.0) and 1 mM EDTA (pH 8.0)] and incubated with MNase [0.1 U/ml; Sigma-Aldrich, N3755; MNase powder was reconstituted with water and adjusted to 50% glycerol to achieve MNase solution (0.1 U/μl) and stored at −20°C] and 2 mM CaCl_2_ at 37°C for 25 min while shaking at 1000 rpm. The reaction was stopped by adding EGTA to a final concentration of 10 mM. The cells were pelleted at 1000*g* for 2 min at 4°C, washed with 5 ml of cold TE, pelleted again, and resuspended in 4 ml of cold TE. The cells were lysed by ultrasound using a Branson sonicator (nine cycles of 20-s ON followed by 40-s OFF with 40% amplitude in ethanol-ice bath). The cell lysates were adjusted to 5 ml of radioimmunoprecipitation assay (RIPA) buffer by sequential addition of Triton X-100 to 1%, sodium deoxycholate (DOC) to 0.1%, NaCl to 140 mM and SDS to 0.1%, and TE to 1x, incubated at 4°C for 10 min on a rotating wheel, and centrifuged for 5 min at 12,000*g* to remove insoluble residues. Soluble cell lysates (cross-linked chromatins) were aliquoted, frozen in liquid nitrogen, and stored at −80°C. A 100-μl aliquot of each cell lysate was used to purify the DNA and estimate the DNA content.

#### Preparation of MNase-digested chromatin from cross-linked Drosophila embryos

Approximately 0.7 g of 16- to 18-hour-old *Drosophila* embryos was dechorionized by incubation in 3 to 2.5% Na-hypochlorite solution for 3 min at room temperature, washed twice with 0.4% NaCl and 0.03% Triton X-100, and cross-linked with 1.8% formaldehyde solution in 50 mM Hepes (pH 7.6), 1 mM EDTA (pH 8.0), 0.5 mM EGTA (pH 8.0), and 100 mM NaCl by shaking for 20 min at room temperature in the presence of *n*-heptane. The reaction was stopped by adding glycine (pH 7.0) to a final concentration of 0.125 M. Cross-linked embryos were washed once in cold ChIP wash buffer A for 10 min at 4°C and once in cold ChIP wash buffer B for 10 min at 4°C. The cross-linked embryos were resuspended in 5 ml of cold ChIP wash buffer B, transferred into a 7-ml homogenizer, and disrupted by several strokes of a tight pestle. The cross-linked embryonic cells were pelleted at 2000*g* for 2 min at 4°C, washed once with 5 ml of cold TE buffer, resuspended in 3.5 ml of cold TE buffer, and incubated at 37°C with MNase (0.1 U/ml) and 2 mM CaCl_2_ for 25 min while shaking at 1000 rpm. The reaction was stopped by adding EGTA to a final concentration of 10 mM. The cross-linked embryonic cells were precipitated by centrifugation at 1000*g* for 2 min at 4°C, washed with 5 ml of cold TE, precipitated again, and resuspended in 3 ml of cold TE. The cells were lysed by ultrasound using a Branson sonicator (nine cycles of 20-s ON followed by 40-s OFF with 30% amplitude in ethanol-ice bath). The resulting lysates were adjusted to 3.5 ml of RIPA by sequential addition of Triton X-100 to 1%, DOC to 0.1%, NaCl to 140 mM and SDS to 0.1%, and TE to 1x and processed as described above for the cross-linked cultured cell lysates.

#### Immunoprecipitation

ChIP was done essentially as described in (*6*) with the following modifications. A standard amount of cross-linked cell lysates containing either 100 μg of DNA (for cultured cells) or 50 μg of DNA (for embryos) was used for immunoprecipitations in a total volume of 500 μl of RIPA buffer [140 mM NaCl, 10 mM tris-HCl (pH 8.0), 1 mM EDTA, 1% Triton X-100, 0.1% SDS, and 0.1% DOC]. The resulting immunoprecipitated DNA was dissolved in either 400 μl of water for qPCR analysis or in 40 μl of water for ChIP-seq library preparation. The nucleotide sequences of primers used for qPCR analysis are listed in table S3. Serial dilutions of DNA isolated from cross-linked cell lysates (input) were used to make the standard curves, and the abundance of specific regions in the immunoprecipitated DNA was expressed as a fraction of input. Rabbit polyclonal antisera against RING1 and Psc were raised by immunization with recombinant GST fusion proteins containing amino acid residues 150 to 280 of RING1 and amino acid residues 821 to 1021 of Psc, respectively. The antibodies were further affinity purified as described in (*86*) and are available for purchase from Agrisera AB. All other antibodies were purchased from vendors listed in table S2.

### ChIP sequencing

Four nanograms of ChIP DNA and the corresponding input DNA was used to prepare sequencing libraries. The library list and the corresponding fractions of the total material used for preparation are indicated in table S4. The libraries were prepared using the NEBNext Ultra II FS DNA Library Prep with Beads (New England Biolabs, #E6177S) according to the manufacturer’s instructions, with minor modifications described below. To get the libraries of fragments with an average size of ~350 to 400 bp, the DNA samples were fragmented for 10 min at 37°C using the NEBNext Ultra II FS Enzyme Mix from the kit. The Illumina adaptor was diluted 25-fold before the ligation to the DNA fragments. The libraries were multiplexed using the NEBNext Multiplex Oligos for Illumina, Set 1 (New England Biolabs, #E7335S) and Set 2 (New England Biolabs, #E7500S) during nine cycles of amplification and cleaned up twice with the supplied magnetic beads to remove the primers and adaptor dimers. A fraction of each library was used to prepare 10 μl of 10 nM solution. All individual 10 nM libraries were combined proportionally to one 10 nM library pool. Fractions of each ChIP DNA and input DNA used to prepare 10 nM library are listed in table S4. The pool was sequenced on one lane of the 10B flow cell using the NovaSeq X Plus system and XLEAP-SBS sequencing chemistry (Illumina).

### ChIP-seq data analysis

#### Read alignment

Sequencing reads were aligned to the *D. melanogaster dm6* reference genome using bowtie2 (v2.4.4) (*87*) with arguments set to –phred33 --no-discordant --very-sensitive-local -p 32. The alignment output was further filtered using samtools (v1.3.1) to remove reads with a mapping quality score less than 30 (-h -b -@ 32 -q 30). The resulting filtered BAM files were used as input to generate bedGraph profiles using the MACS3 (*88*) pileup command with the BAMPE output option. Aligned reads were visualized with IGB (v.10.1.0) (*89*). Distribution of ChIP-seq signals at the whole chromosome scale was visualized with the R package chromoMap v0.3 (*90*).

#### ChIP-seq signal normalization and filtering

For quantitative comparison of ChIP-seq signals for the same antibody from cells with different genetic backgrounds, we adopted the siQ-ChIP proposed by Dickson and colleagues (*50*, *51*). siQ-ChIP aims to estimate ChIP yields (*Y*) for every genomic position. It postulates that ChIP yield *Y*(*i*) for the genomic position *i* corresponds to the number of DNA fragments recovered in a ChIP reaction that overlap this position *R_IP_*(*i*)

$$Y\left(i\right)=R_{\mathrm{IP}}\left(i\right)$$ (1)

*R_IP_*(*i*) could be determined by sequencing all DNA molecules recovered in a ChIP reaction. In practice, it is customary to sequence just a fraction of all DNA immunoprecipitated in one reaction, and often only the nucleotide sequences of the ends of the immunoprecipitated DNA fragments are determined. Therefore, typically, the sequencing assay provides the information on the number of sequence tags *R′_IP_*(*i*) that overlap position *i* rather than *R_IP_*(*i*). *R_IP_*(*i*) is proportional to *R′_IP_*(*i*), and the two values are connected by the scaling coefficient *S_IP_*. This coefficient depends on several factors, for example, sequencing depth, the fraction of the library taken for sequencing, and the fraction of the ChIP DNA used to prepare the library. Considering the above, we can rewrite Eq. 1 as follows

$$Y\left(i\right)=S_{\mathrm{IP}}\times R_{\mathrm{IP}}^{\prime }\left(i\right)$$ (2)

We posit that, under the standard conditions [i.e., all ChIP reactions were performed with the same amount of antibody and chromatin lysate, the immunoprecipitated DNA was uniformly fragmented before library construction, and all sequencing libraries were adjusted to the same concentration (10 nM) and sequenced in parallel as part of the same pool], *S_IP_* is the same for all genomic positions and can be calculated as follows

$$S_{\mathrm{IP}}=\frac{10^{6}}{R_{\mathrm{IP}}^{\prime }\times F_{\text{proc}}^{\mathrm{IP}}\times F_{\mathrm{lib}}^{\mathrm{IP}}}$$ (3)

where *R′_IP_* is the total number of sequence tags obtained from sequencing of the corresponding library, *F_proc_* is a fraction of the ChIP DNA used to prepare the sequencing library, *F_lib_* is a fraction of the sequencing library used to prepare the 10 nM library solution, and the numerator 10^6^ scales the yields to 1 million sequenced reads. Combining Eqs. 2 and 3 allows us to express the estimated ChIP yields *Y*(*i*) for any genomic position *i* as a function of variables directly measured in the assay

$$Y\left(i\right)=\frac{R_{\mathrm{IP}}^{\prime }\left(i\right)}{R_{\mathrm{IP}}^{\prime }\times F_{\text{proc}}^{\mathrm{IP}}\times F_{\mathrm{lib}}^{\mathrm{IP}}\times 10^{-6}}$$ (4)

Thus, for each ChIP sample, the $F_{\text{proc}}^{\mathrm{IP}}$, $F_{\mathrm{lib}}^{\mathrm{IP}}$ values were recorded during library preparation, and the total number of sequenced reads *R′_IP_* was reported after sequencing. The corresponding values are indicated in table S4. The *R′_IP_*(*i*) values were calculated using bedtools (v2.30.0) (https://bedtools.readthedocs.io) using a two-step procedure. First, the genome was segmented into 50-bp bins using the bedtools makewindows -w 50 command. Second, the number of sequence tags for each bin *i* was calculated using the bedtools map -o mean command.

For consistency, the read distributions obtained after sequencing DNA from the corresponding chromatin input materials were scaled as described above. Although in this case, the resulting values have no biological meaning. For all the downstream analyses and visualization, the normalized ChIP-seq signals (i.e., genomic distributions of estimated ChIP yields) were filtered to remove spurious peaks corresponding to highly repeated genomic regions. To this end, we removed all 50-bp bins whose scaled read counts in the corresponding chromatin input dataset exceeded the mean plus 3 SDs for this dataset.

#### Definition of bound regions

Regions significantly enriched by ChIP with anti-Psc and anti-RING1 antibodies were identified separately for each replicate experiment using the MACS3 callpeak command with the following parameters: -f BAMPE -g dm --qvalue 0.1. Only regions with fold enrichment (FC) ≥ 3 were used for further analyses. Regions significantly enriched by ChIP with anti-H3K27me3 antibodies were defined using Epic2 software (*91*) with the genome parameter set to --genome dm6. Similarly, only regions with FC ≥ 3 were used for further analyses. PREs were defined as regions significantly enriched by anti-RING1, anti-Psc, and anti-H3K27me3 antibodies using bedtools intersect function. For all H2AK118ub ChIP-seq datasets, 1-kb windows with normalized ChIP-seq signals exceeding 3 SDs from the mean normalized signal of ChIP-seq assays with chromatin from RING1-KO cells were considered significantly enriched.

#### Genome segmentation by k-means clustering

The genome was divided into 1-kb windows using bedtools makewindows -w 1000, and each window was assigned a mean normalized ChIP-seq signal value computed for H2AK118ub signals from ChIP-seq assays with chromatins from the control, Psc/Su(z)2-KO, and Siesta-KO cells. The mean normalized H2AK118ub ChIP-seq signal values for each genetic background were scaled using the scale function to standardize the data across samples. These scaled values were then used to segregate the 1-kb windows into three groups by unsupervised *k*-means clustering using the stats package (v.4.4.3) (https://R-project.org/) and the following parameters: centers = 3 and nstart = 25. The 1-kb windows with associated ChIP-seq signal values and cluster assignments are listed in the supplementary file S1.

### Thousand Reporters Integrated in Parallel

#### Cell transfection

The control (Ras17), Psc/Su(z)2-KO, Siesta-KO, and dRING-KO cells were cultured at 25°C in Schneider’s medium (BioConcept) supplemented with 10% of heat-inactivated fetal bovine serum (Sigma-Aldrich), streptomycin (0.1 mg/ml), and penicillin (100 U/ml) (Gibco) under sterile conditions. For transfection, 2 × 10^6^ cells were seeded in a 6-well plate 1 day before the procedure. Transfection was performed using X-tremeGENE HP DNA Transfection Reagent (Sigma-Aldrich) according to the manufacturer’s instructions. Cells of one well were transfected with 2 μg of the equimolar DNA mixture of the pPB-MtnA-eGFP-PI-14-BC-libr, pPB-MtnA-eGFP-PI-16-BC-libr, pPB-Hsp70-eGFP-PI-11-BC-libr, and pPB-Hsp70-eGFP-PI-3-BC-libr (supplementary files S2 to S5) barcoded plasmid libraries and 0.4 μg of the DNA of the construct to express the piggyBac transposase fused to histone H1 under control of the *Hsp70Bb* promoter (phsp70-pBac-H1; supplementary file S6). Cultures of cells transfected with mixtures lacking the construct expressing the piggyBac transposase (mock-transfected) and nontransfected cells were grown in parallel. Twenty-four to 48 hours after transfection, the treated and control cell cultures were subjected to a heat shock at 37°C for 2 hours to induce the transposase expression. The cell cultures were allowed to grow for 3 to 4 days, after which several aliquots of ~10,000 cells were subcultured to establish TRIP cell pools, each containing a unique collection of integrated transgenes. Each TRIP cell pool was further cultured for a minimum of 1 month to eliminate the transfected DNA that did not integrate into the genome.

#### GFP induction with CuSO_4_

To induce transcription of the transgenic *GFP*, 3 × 10^6^ cells plated in triplicate 2 days earlier were treated with 0.5 mM CuSO_4_ for 2 hours at 25°C. The control cell cultures were grown in the same way, but without the addition of supplementary CuSO_4_.

#### RNA isolation and cDNA synthesis

For each replicate preparation, 1 × 10^7^ cells were collected by centrifugation and lysed in 1 ml of TRIzol reagent (Invitrogen, #15596026). The RNA was isolated according to the manufacturer’s instructions. Two micrograms of the RNA was used for cDNA synthesis with oligo(dT)18 primer by the RevertAid H Minus First Strand cDNA Synthesis Kit (Thermo Fisher Scientific, #K1631). The cDNA was treated with RNase A to remove the RNA template, cleaned up with the Zymo DNA purification kit (#D4034), and eluted with 30 μl of 0.1x TE.

#### TRIP-mapping library preparation

Two micrograms of genomic DNA from each cell line was digested with Mbo I at 37°C for 6 hours, followed by incubation at 65°C to inactivate the enzyme. Digested DNA (one-third of the reaction volume, 0.7 μg) was circularized in a 400-μl reaction with 20 U of T4 DNA ligase [in the presence of 1 mM adenosine triphosphate (ATP), 10 mM MgCl_2_, 1 mM DTT, and 60 mM tris-HCl (pH 7.4)] at +4°C for 16 hours followed by incubation at 65°C for 10 min to inactivate the ligase. The resulting circular DNA was purified by phenol-chloroform extraction and dissolved in 30 μl of water. Five microliters of purified circular DNA solution was used for the first round of inverse PCR to amplify the parts that included the TRIP barcodes and adjacent genomic DNA from insertion sites. One microliter of the resulting PCR product was used in the second PCR round to introduce sequencing indexes. One microliter of the resulting product from the second PCR round was used in the third round of PCR to incorporate Illumina sequencing adaptors. The list of primers and amplification conditions is indicated in table S5. The resulting TRIP-mapping libraries were cleaned up twice with SPRIselect magnetic beads (Beckman Coulter), adjusted to 10 nM, and pooled proportionally into the 10 nM TRIP-mapping pool. All sequenced TRIP-mapping libraries are listed in table S6.

#### TRIP-normalization library and TRIP-cDNA library preparation

A total of 2.5 × 10^7^ cells were lysed overnight in 1x TE with Proteinase K (0.5 mg/ml) and 0.5% SDS, followed by phenol-chloroform extraction and ethanol precipitation. The resulting genomic DNA was dissolved in 1x TE [10 mM tris-HCl (pH 8.0) and 1 mM EDTA] and treated with RNase A (0.1 mg/ml) at 37°C for 30 min. RNase A was removed by phenol-chloroform extraction, and the DNA was ethanol precipitated and dissolved in 1x TE.

One hundred nanograms of the purified genomic DNA or 10% of the cDNA product from the synthesis reaction (described above) was used for normalization and cDNA library preparation, respectively. The DNA was subjected to two rounds of PCR to introduce sequencing indexes and Illumina adaptors for sequencing. PCR products were cleaned up with SPRIselect magnetic beads after every PCR round and eluted with 25 μl of 0.1x TE buffer. Five microliters of the eluted DNA after the first PCR round was used in the second round. The libraries were adjusted to 10 nM and mixed proportionally into TRIP-normalization and TRIP-cDNA pools. The libraries are listed in table S6. The primers and amplification conditions are described in table S7.

Fractions of TRIP-mapping, TRIP-normalization, and TRIP-cDNA libraries were combined into a single 10 nM pool at ratios 1:2:2. The pool was sequenced on one lane of the 10B flow cell using the NovaSeq X Plus system and XLEAP-SBS sequencing chemistry (Illumina).

### TRIP data analyses

As illustrated in Fig. 6, the TRIP assay yields three types of sequencing reads. Although all libraries were sequenced as paired end, for the “expression” and “normalization” datasets, only single-end reverse 150-bp reads were used. These reads, which contained the plasmid region and a unique barcode, were used to count the barcode frequencies in genomic DNA and cDNA samples, respectively. The third dataset contains “mapping” reads. These are paired-end 150-bp reads obtained by sequencing of inverse PCR products. The “mapping” reads include the 5′ end of the piggyBac transposon and a neighboring genomic DNA sequence in the forward read, as well as a barcode and a genomic sequence adjacent to the Dpn II site in the reverse read.

The initial fastq file with reads obtained after sequencing the TRIP library pool was parsed according to the sequencing index (table S6) using sabre software (https://anaconda.org/bioconda/sabre) and then by the promoter index using the awk-based bash script (supplementary file S7). The parsed fastq files with “normalization,” “expression,” and “mapping” reads were grouped by individual TRIP experiment and processed with TRIP Analysis Software Kit (TASK) software (*92*) with parameter and read structure settings described in supplementary file S8. The example command line instructions to process a dataset with TASK software are provided in supplementary file S9.

The barcode lists returned by TASK were filtered to remove the instances that may have arisen due to nucleotide substitutions during the PCR steps of library preparation. To this end, the following algorithm was used. First, the barcode instances were sorted according to their counts in the “normalization” sample. Second, the most frequent barcodes were ranked the highest, and for each highest-ranked barcode instance, “mutant” versions, defined as barcodes with distinct nucleotide sequences appearing at the same position (pos_r), were identified and removed. Third, the resulting lists were filtered to remove all barcode instances with a mapping quality score less than 10 (mapq_r > 10). Last, the identical barcode instances associated with the same genomic position in two biologically independent replicate experiments were deemed to represent genuine transgenic insertions.

Each genuine transgenic insertion was assigned the TRIP signal calculated as follows

$$\text{TRIP signal}=\frac{\text{barcode count}\in \text{expression sample }\left(\mathrm{rpm}\right)}{\text{barcode count}\in \text{normalization sample}\;(\mathrm{rpm})}$$

The list of genuine transgenic insertions with associated nucleotide sequence, genomic position, TRIP signal, etc., is reported in supplementary file S10.

### Statistical analysis and plotting

All statistical analyses were performed using R (https://R-project.org/), and plots were generated using ggplot2 (*93*).
