# Supplementary material for: Polycomb repression works without Siesta, the Drosophila ortholog of mammalian PCGF3
Source: Sci Adv. 2026 Mar 6;12(10):eaec0733. doi: 10.1126/sciadv.aec0733 (PMC12965321; doi:10.1126/sciadv.aec0733)
Supplement: Supplementary file 1 — Figs. S1 to S14 Tables S1 to S7 Legends for movies S1 to S4 Legends for files S1 to S10 [file sciadv.aec0733_sm.pdf]

Science Advances
AAAS

Supplementary Materials for

Polycomb repression works without Siesta, the *Drosophila* ortholog of mammalian PCGF3

Tatyana G. Kahn *et al.*

Corresponding author: Yuri B. Schwartz, yuri.schwartz@umu.se

Sci. Adv. **12**, eaec0733 (2026)
DOI: 10.1126/sciadv.aec0733

The PDF file includes:

Figs. S1 to S14
Tables S1 to S7
Legends for movies S1 to S4
Legends for files S1 to S10

Other Supplementary Material for this manuscript includes the following:

Movies S1 to S4
Files S1 to S10

Figure S1

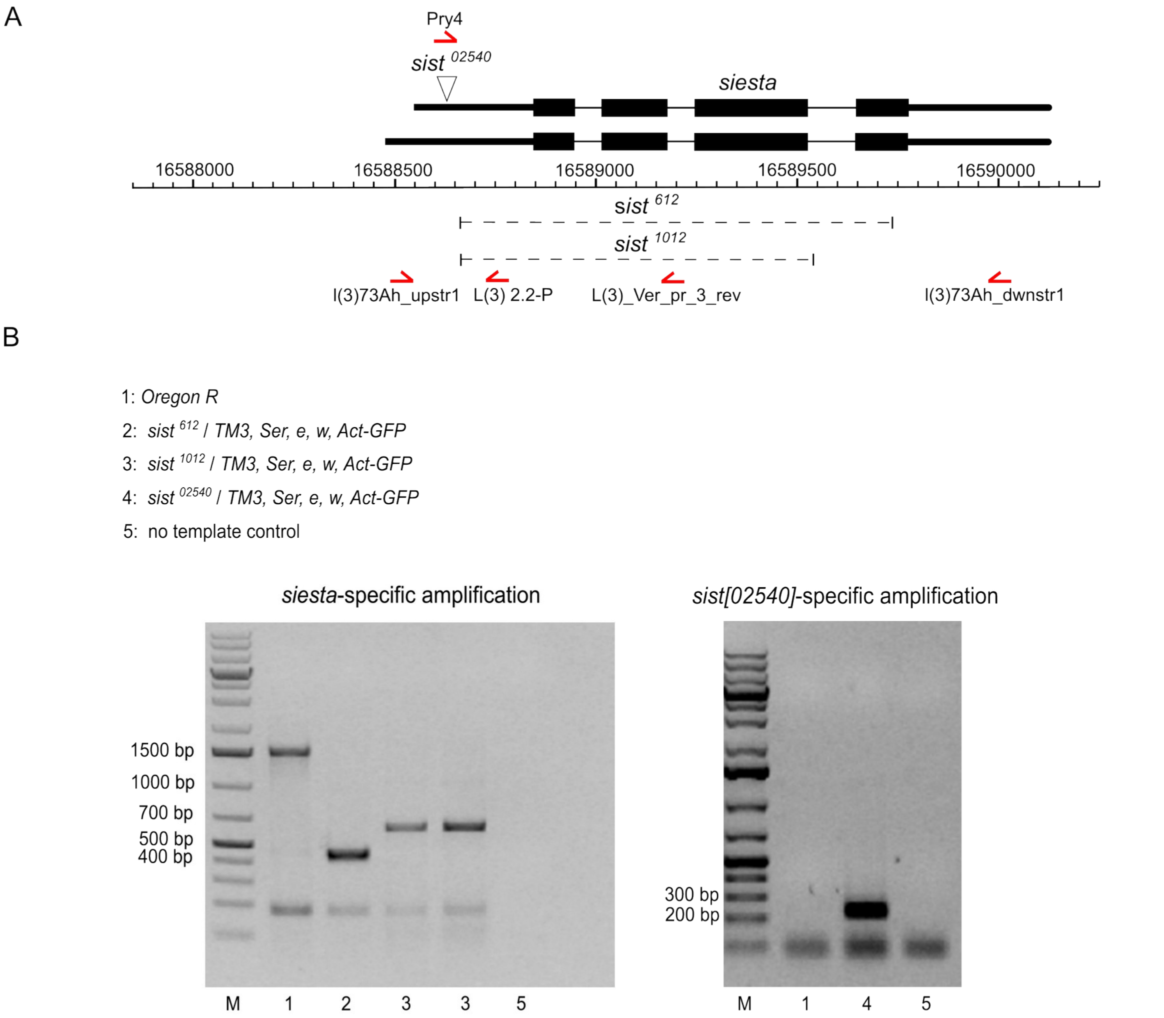

Amplicon	Forward primer	Reverse primer	Product [612] allele	Product [1012] allele	Product wt allele	Product [02540] allele
siesta	l(3)73Ah_upstr1	l(3)73Ah_dwnstr1	426 bp	610 bp	1479 bp	N/A
sist[02540]	Pry4	L(3) 2.2-P	N/A	N/A	No product	234 bp

Figure S1. The structure and PCR genotyping of *Siesta* alleles. A. The schematics of the *siesta* locus. Two alternative transcripts above the coordinate scale (*dm6* genomic release) are shown with Transcription Start Sites (TSS) to the left. Thin lines indicate introns, and black boxes correspond to the coding parts. The position of transposon insertion in the $sist^{02540}$ allele (also known as $l(3)73Ah^{02540}$) is indicated by a white triangle. Dashed lines mark the extent of $sist^{612}$ and $sist^{1012}$ deletions. Red half-arrows indicate positions of PCR primers used for genotyping. **B.** To verify the presence of specific *siesta* alleles, genomic DNA from flies of the indicated genotypes was used as a template for PCR with the primer combinations shown in the table. DNA from Oregon-R strain was used as a control. PCR products were analysed by gel electrophoresis in 1% agarose gel along with the Gene Ruler 1kb Plus molecular weight marker (M).

Figure S2

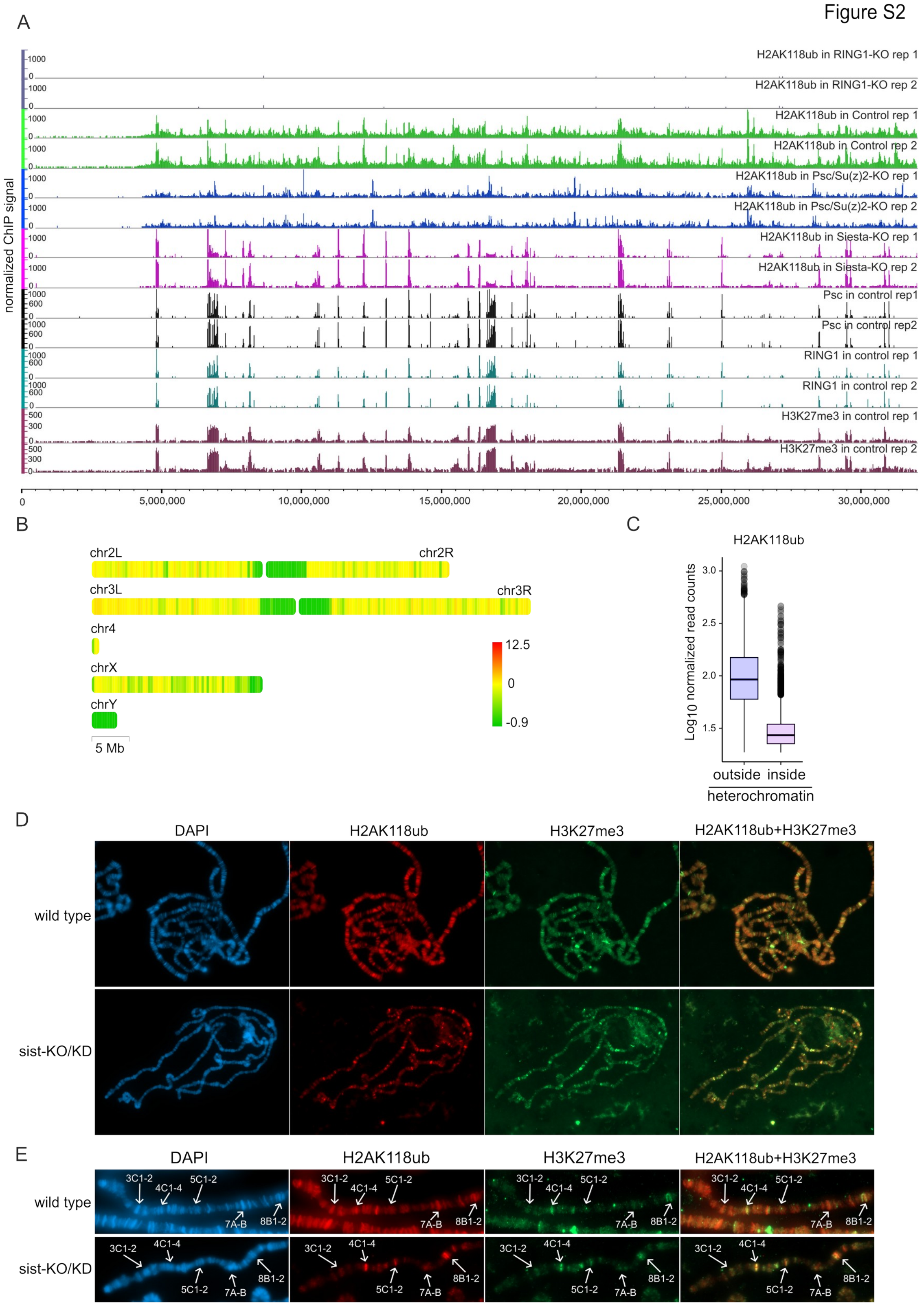

Figure S2. Overview of genomic H2AK118 ubiquitylation. A. Genome browser tracks showing H2AK118ub ChIP-seq profiles across *Drosophila* chromosome 3R in RING1-KO, control, Psc/Su(z)2-KO, and Siesta-KO cell lines (two replicates each). Additional tracks display ChIP-seq profiles for Psc, RING1, and H3K27me3 in control cells (two replicates each). The x-axis indicates

genomic coordinates in *dm6* genomic release scale. **B.** The distribution of H2AK118ub ChIP-seq signal along *Drosophila* chromosomes. Average scaled signal intensities for fixed 350 kb windows tiling the genome were calculated using chromoMap v0.3. These were further converted into Z-scores and displayed as colour gradient ranging from green (low signal) to yellow (intermediate signal) and red (high signal). Note depletion of H2AK118ub from pericentromeric regions of chromosomes 2, 3 and X, as well as heterochromatin chromosome Y. **C.** Boxplots comparing normalized H2AK118ub ChIP-seq signals outside versus inside pericentromeric heterochromatin regions. Boxplots indicate the median and span interquartile range with whiskers extending 1.5 times the range and outliers shown as circles. **D.** Representative pictures of polytene chromosome spreads from wild type (*Oregon R*) and $sist^{612}/sist^{02540}$ (Sist-KO/KD) third instar larvae stained with DAPI and antibodies against H2AK118ub and H3K27me3. The rightmost images show the merge between two immunostainings. **E.** Representative pictures of the distal part of X chromosome from the third instar larvae of the same genotypes. Positions of characteristic polytene chromosome bands according to Bridges nomenclature are marked with white arrows. Note the loss of H2AK118ub signal from the chromosome arms in the Sist-KO/KD mutants except from sites brightly stained with H3K27me3.

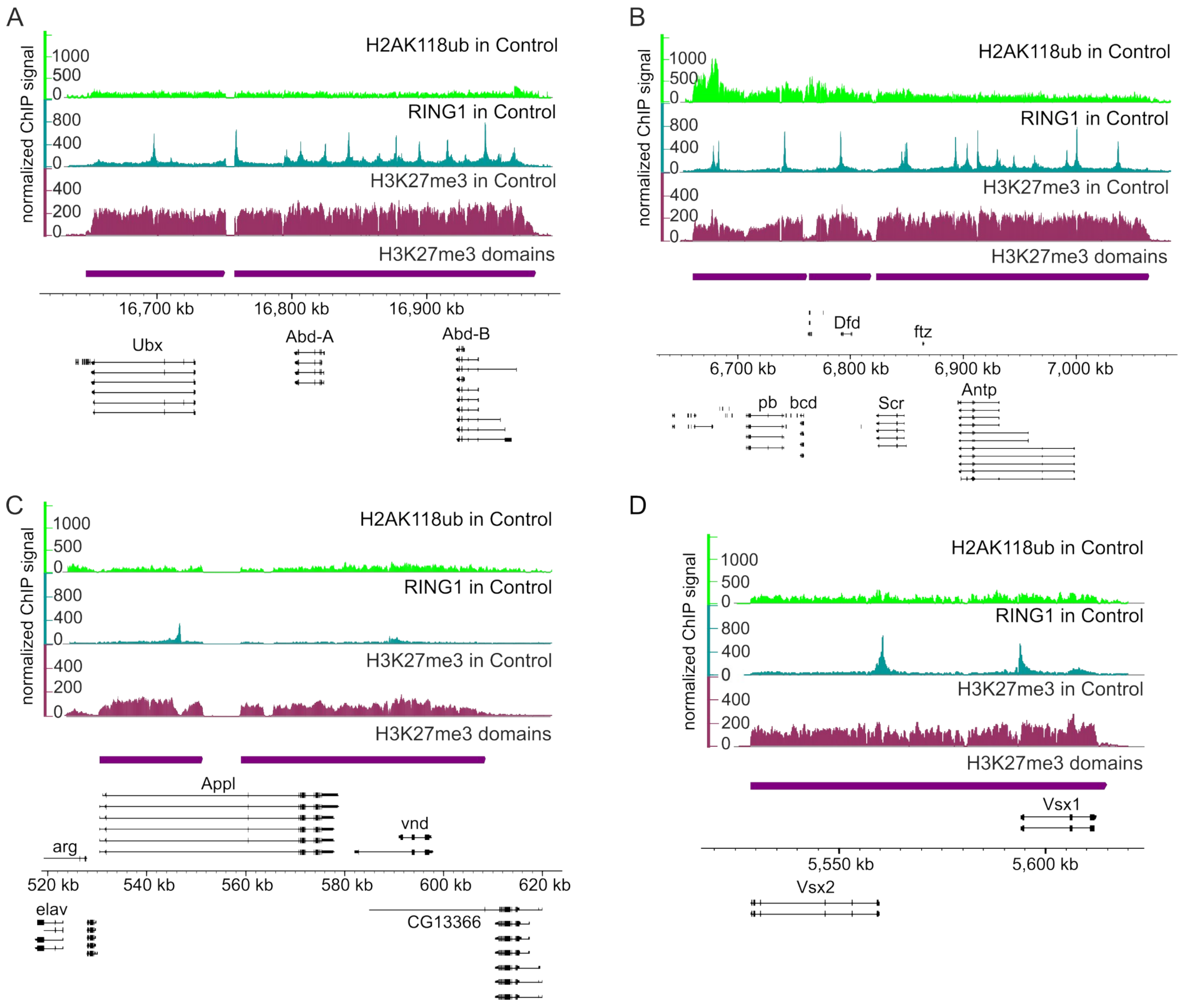

Figure S3. Examples of loci repressed by the Polycomb system with very low H2AK118ub. Genome browser tracks for H2AK118ub, RING1 and H3K27me3 ChIP-seq signals in control cells over the *bithorax complex* (**A**), the *Antennapedia complex* (**B**), *Appl – vnd* locus (**C**) and *Vsx1 – Vsx2* gene cluster (**D**). Genes shown above the coordinates scale (in *dm6* genomic release) are transcribed from left to right, genes shown below the coordinates scale are transcribed from right to left. The extent of H3K27me3 domains used to compare H3K27me3 and H2AK118ub ChIP-seq signals is shown as thick purple lines below the genome browser tracks.

Figure S4

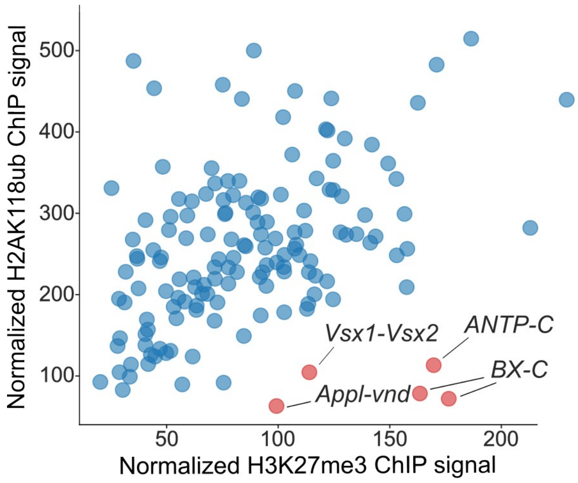

Figure S4. A small number of PRE-equipped genes have very low H2AK118ub compared to H3K27me3. The scatter plot shows averaged normalized ChIP-seq signals within continuous PRE-containing regions in control cells with significantly elevated H3K27me3 ChIP-seq signals (H3K27me3 domains). Each point corresponds to one H3K27me3 domain.

Figure S5

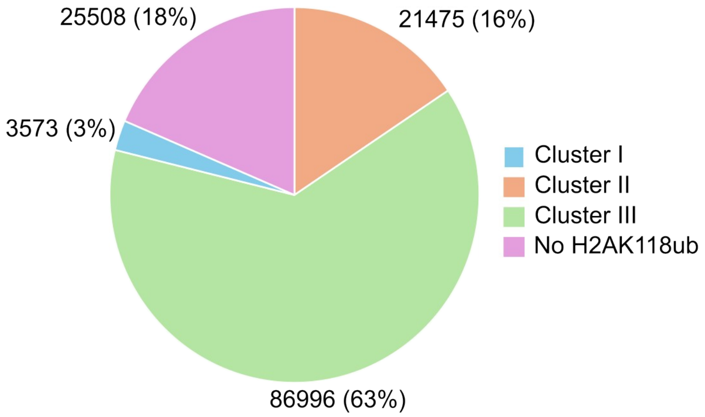

Figure S5. Relative representation of 1kb genomic segments (bins) assigned to four different categories based on comparison of H2AK118ub ChIP-seq signals in the control, Psc/Su(z)2-KO and Siesta-KO cells. An absolute number of 1kb bins in each category and percentile (in parentheses) are shown next to the corresponding segments of the pie chart.

Figure S6

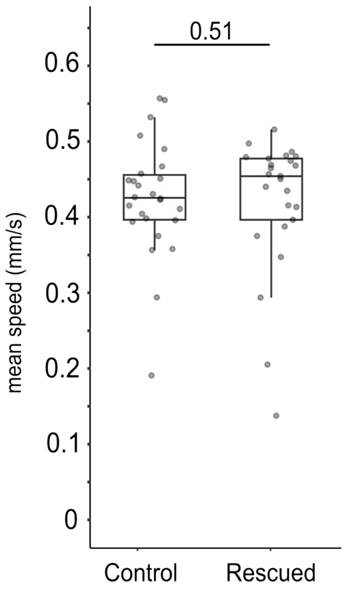

Figure S6. Transgenic expression of the Siesta protein complements the locomotion defect of Siesta-KO mutants. Motion tracking of the wild type Oregon-R (Control) and *sist::Twin-Strep-Myc-sist*; *sist*^{612}/*sist*^{1012} (Rescued) second instar larvae. The box plots show median speeds and span the interquartile range with whiskers extending 1.5 times the range. Grey dots indicate the mean speeds of individual larvae. The difference between the two groups is not significant (Wilcoxon rank sum test, p-value displayed above the box plots).

Figure S7

A

1: *Oregon R*

2: *Sce* ^{I48A}; *Sce* ^{KO}

3: *Sce* ^{I48A}; *Sce* ^{KO} / *TM3, Ser*

4: no template control

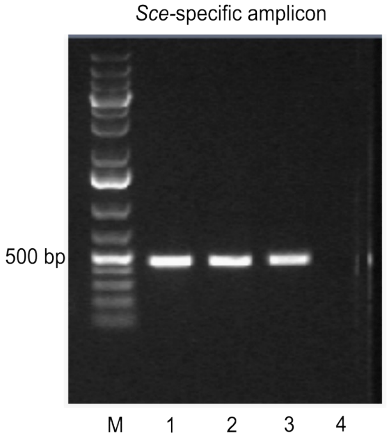

Amplicon	Forward primer	Reverse primer	Product [I48A] transgene	Product wt allele
Sce	Sce_1.1	Sce_1.2	441 bp	441 bp

B

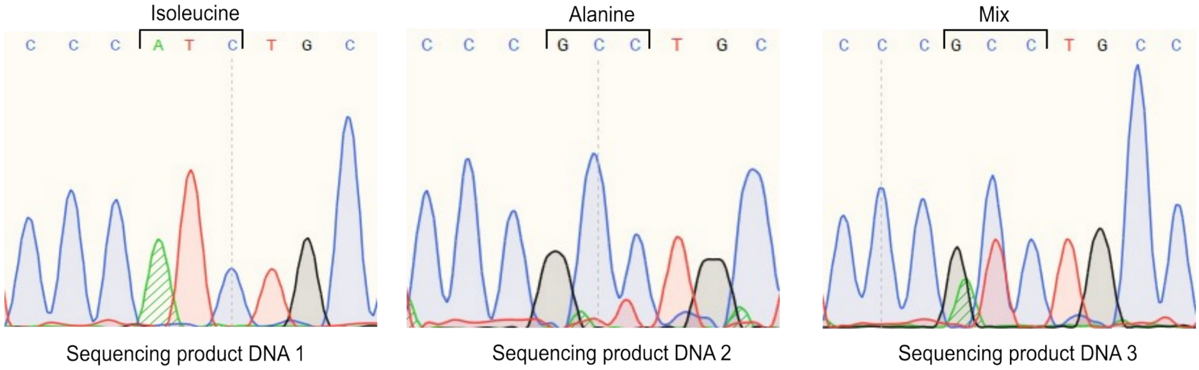

Figure S7. Genotyping of *Sce*^{I48A}*; Sce*^{KO} (RING1-I48A) larvae. A. To verify the presence of *Sce* alleles, genomic DNA from flies of the indicated genotypes was used as a template for PCR using the primer combination shown in the table. DNA from Oregon-R strain was used as a control. PCR products were analysed by gel electrophoresis in 1% agarose gel along with the Gene Ruler 1kb Plus molecular weight marker (M). **B.** Chromatograms of sequencing reactions with PCR products from (**A**) show the Isoleucine to Alanine substitution at position 48 of the transgenic *Sce*.

Figure S8

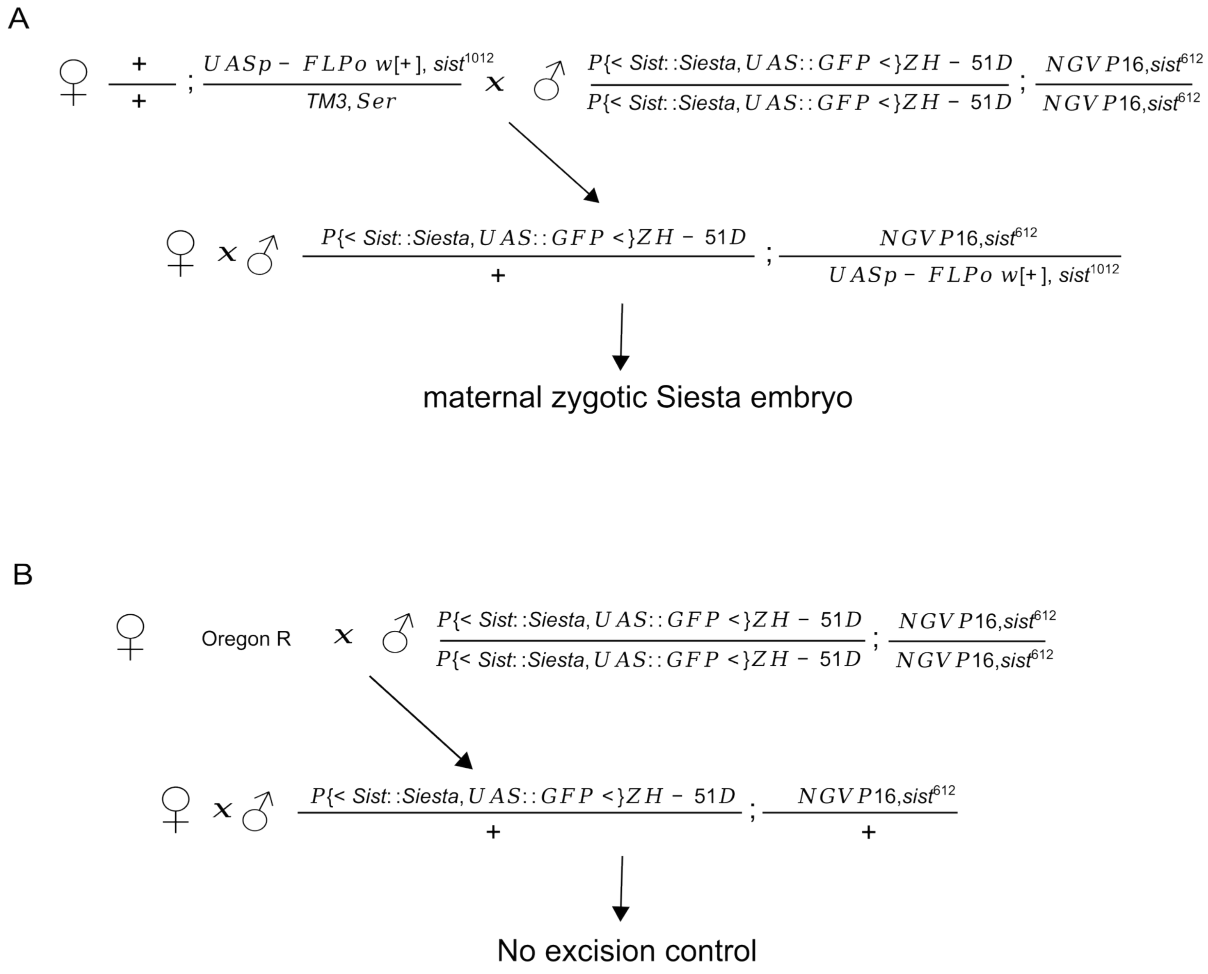

Figure S8. Generation of embryos that lack maternal and zygotic Siesta protein. A. Siesta KO^{mz} embryos were generated as follows. The *P{<sist::siesta, UAS::GFP<}ZH-51D; P{*w^{+mC} *= GAL4::VP16-nos.UTR}*$CG6325^{MVD1}$*,* $sist^{612}$ males were crossed to the w^-/w^-*; +/+;* $sist^{1012}$*, {UASp-FLPo,* w^+*}VK33,* y^+*/TM3, Ser, {Act::GFP,* w^+*}, e* females. From the resulting F1 progeny, males and females of the *P{<sist::siesta, UAS::GFP<}ZH-51D/+; P{*w^{+mC} *= GAL4::VP16-nos.UTR}* $CG6325^{MVD1}$*,* $sist^{612}/sist^{1012}$*, {UASp-FLPo,* w^+*}VK33,* y^+ genotype were crossed with each other, which resulted in embryos that lack maternal and zygotic Siesta protein. **B.** Control embryos were generated by crossing Oregon R females with *P{<sist::siesta, UAS::GFP<}ZH-51D; P{*w^{+mC} *= GAL4::VP16-nos.UTR}*$CG6325^{MVD1}$*,* $sist^{612}$ males. The F1 progeny were then crossed with each other. Since there was no FLP recombinase source, the transgenic cassette was not excised.

1: *Oregon R*

2: *Sist::Siesta,UAS::GFP ; NGVP16, sist* ^{612}

3: + ; *UAS::FLP, sist* ^{1012} */ TM3, Ser, Act::GFP, w[+]*

4: *Sist::Siesta,UAS::GFP; sist* ^{612} */ sist* ^{1012}

5: *Sist::Siesta,UAS::GFP*; + / +

6: *lf / CyO ; NGVP16*

7: + ; *UAS::FLP, w[+]*

8: no template control

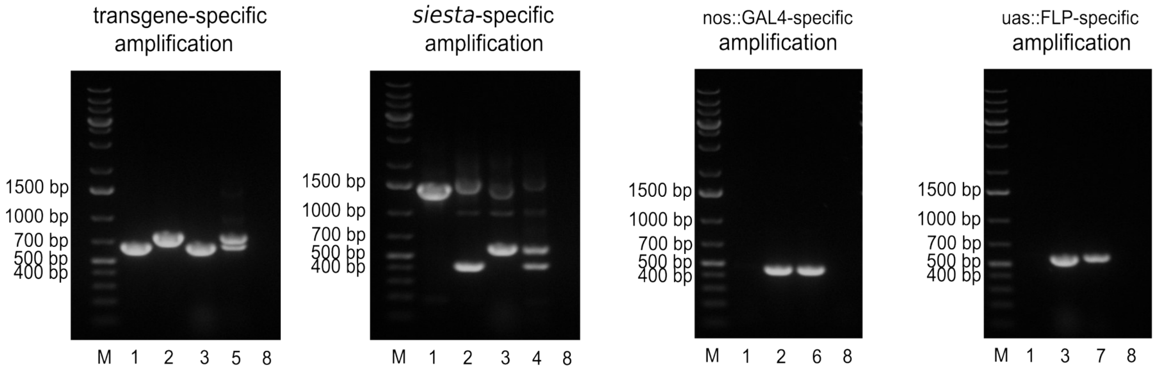

Amplicon	Forward primer	Reverse primer	Product [612] allele	Product [1012] allele	Product wt allele	Product transgene allele
transgene	l(3)73Ah_upstr1	l3_Ver_pr_3_rev	No product	No product	663 bp	782 bp
siesta	l(3)73Ah_upstr1	l(3)73Ah_dwnstr1	426 bp	610 bp	1479 bp	1596 bp

Amplicon	Forward primer	Reverse primer	Product FLP allele	Product NGVP16 allele
nos::GAL4	osGAL4:DBD-fw	CP190-GAL4:DBD-rev	N/A	442 bp
uas::FLP	FLP_For	FLP_Rev	568 bp	N/A

Figure S9. Genotyping of Drosophila strains involved in the generation of embryos lacking maternal and zygotic Siesta protein. DNA from flies of the indicated genotypes was used as a template for PCR using the primer combination shown in the tables. DNA from Oregon-R strain was used as a control. PCR products were analysed by gel electrophoresis in 1% agarose gel along with the Gene Ruler 1kb Plus molecular weight marker (M).

Figure S10

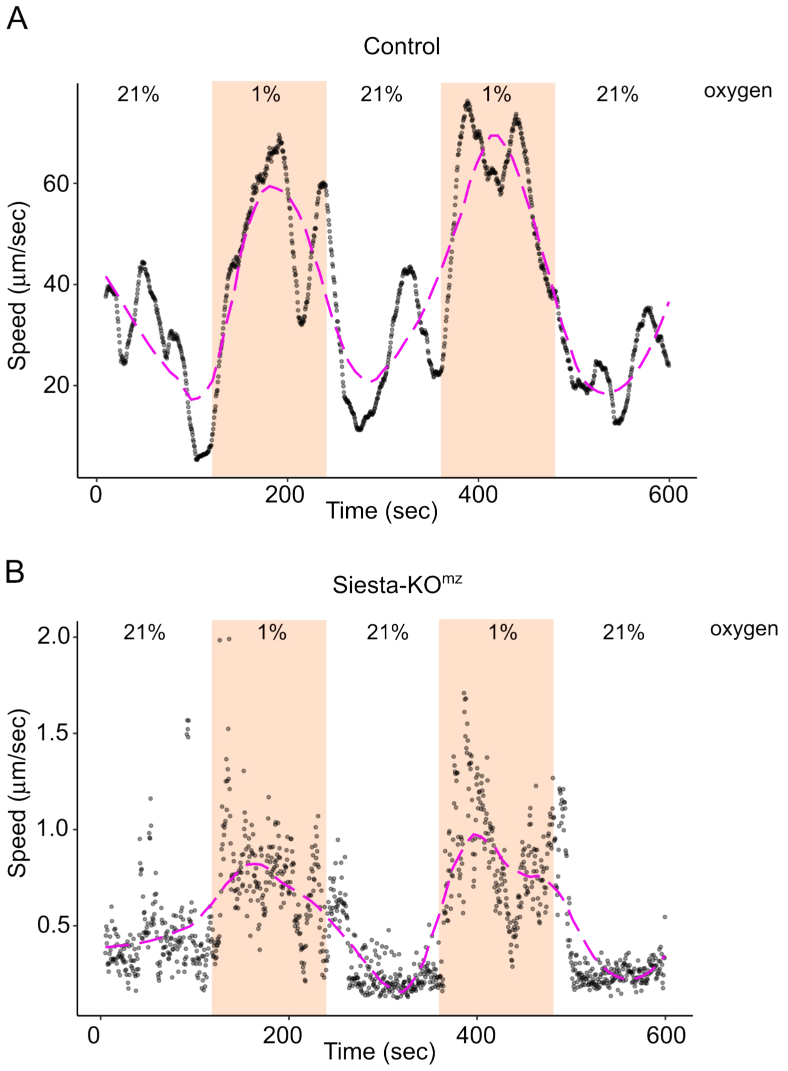

Figure S10. Siesta mutants respond to hypoxia. Motion tracking of control (**A**) and Siesta-KO^{mz} (**B**) first instar larvae under variable oxygen concentrations. The dots indicate the mean speed of all larvae in the camera view field at a given time point. The magenta dashed lines represent the outcome of data fitting with LOESS regression to demonstrate the general trend in speed changes. Note the different y-axis scales for the control and mutant larvae. Both control and Siesta-KO^{mz} larvae increase their crawling speed when exposed to 1% oxygen.

Figure S11

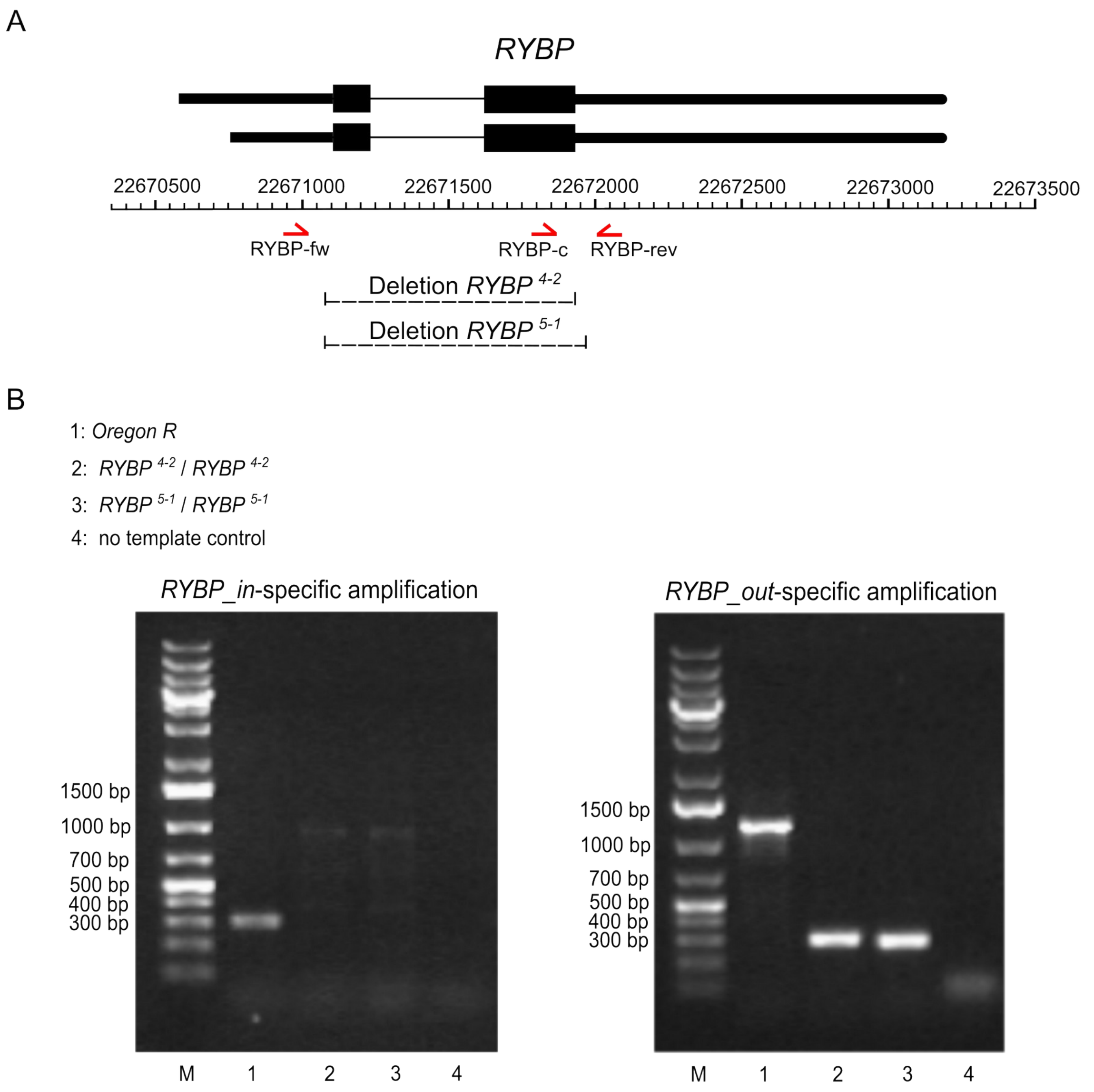

Amplicon	Forward primer	Reverse primer	Product *RYBP*$^{4-2}$ allele	Product *RYBP*$^{5-1}$ allele	Product wt allele
RYBP_in	RYBP-c	RYBP-rev	No product	No product	314 bp
RYBP_out	RYBP-fw	RYBP-rev	312 bp	290 bp	1192 bp

Figure S11. The structure and PCR genotyping of new *RYBP* alleles. A. The schematics of the *RYBP* locus. Two alternative transcripts above the coordinate scale (*dm6* genomic release) are shown with Transcription Start Sites (TSS) to the left. Thin lines indicate introns, and black boxes correspond to the coding parts. Dashed lines mark the extent of *RYBP*$^{4-2}$ and *RYBP*$^{5-1}$ deletions. Red half-arrows indicate positions of PCR primers used for genotyping. **B.** To verify the presence of specific *RYBP* alleles, genomic DNA from flies of the indicated genotypes was used as a template for PCR with the primer combinations shown in the table. DNA from Oregon-R strain was used as a control. PCR products were analysed by gel electrophoresis in 1% agarose gel along with the Gene Ruler 1kb Plus molecular weight marker (M).

Figure S12

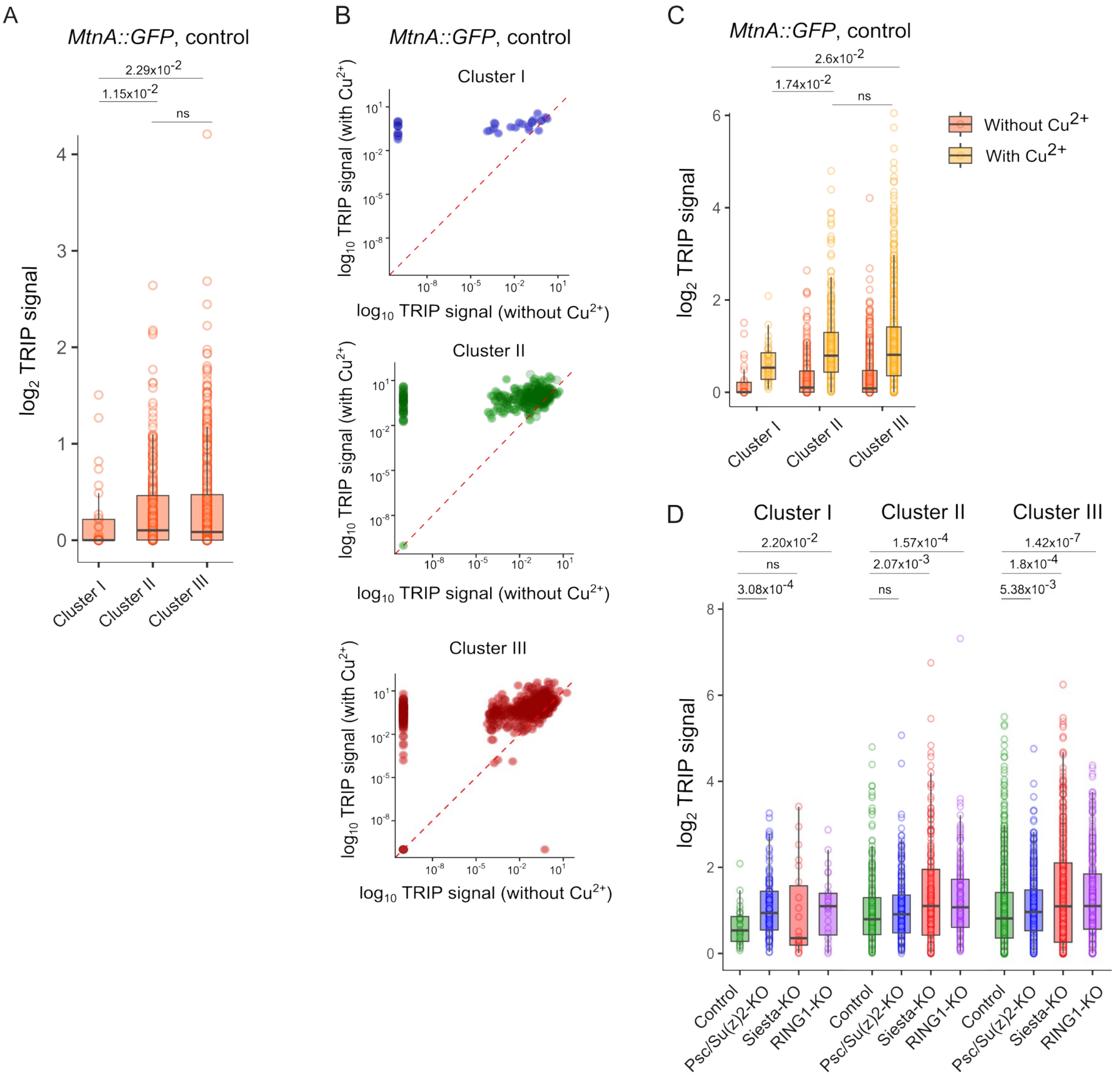

Figure S12. TRIP reveals no negative correlation between transcription and H2AK118ub. A. TRIP signals for the *MtnA::GFP* transgenes integrated in three types of genomic regions in control cells. Here and in **C** and **D**, the boxplots indicate the median and span interquartile range with whiskers extending 1.5 times the range. The differences in medians between corresponding groups were tested for statistical significance using the Wilcoxon rank sum test, and p-values are displayed above the box plots. **B.** Log-log comparison of TRIP signals for the *MtnA::GFP* transgenes before and after copper Cu^{2+} induction in three types of genomic regions in control cells. A dashed red diagonal line (slope = 1) denotes equal signal before and after induction. **C.** TRIP signals for the *MtnA::GFP* transgenes integrated into three types of genomic regions in control cells before and after copper Cu^{2+} induction. **D.** TRIP signals for the *MtnA::GFP* transgenes in different cell lines.

Figure S13

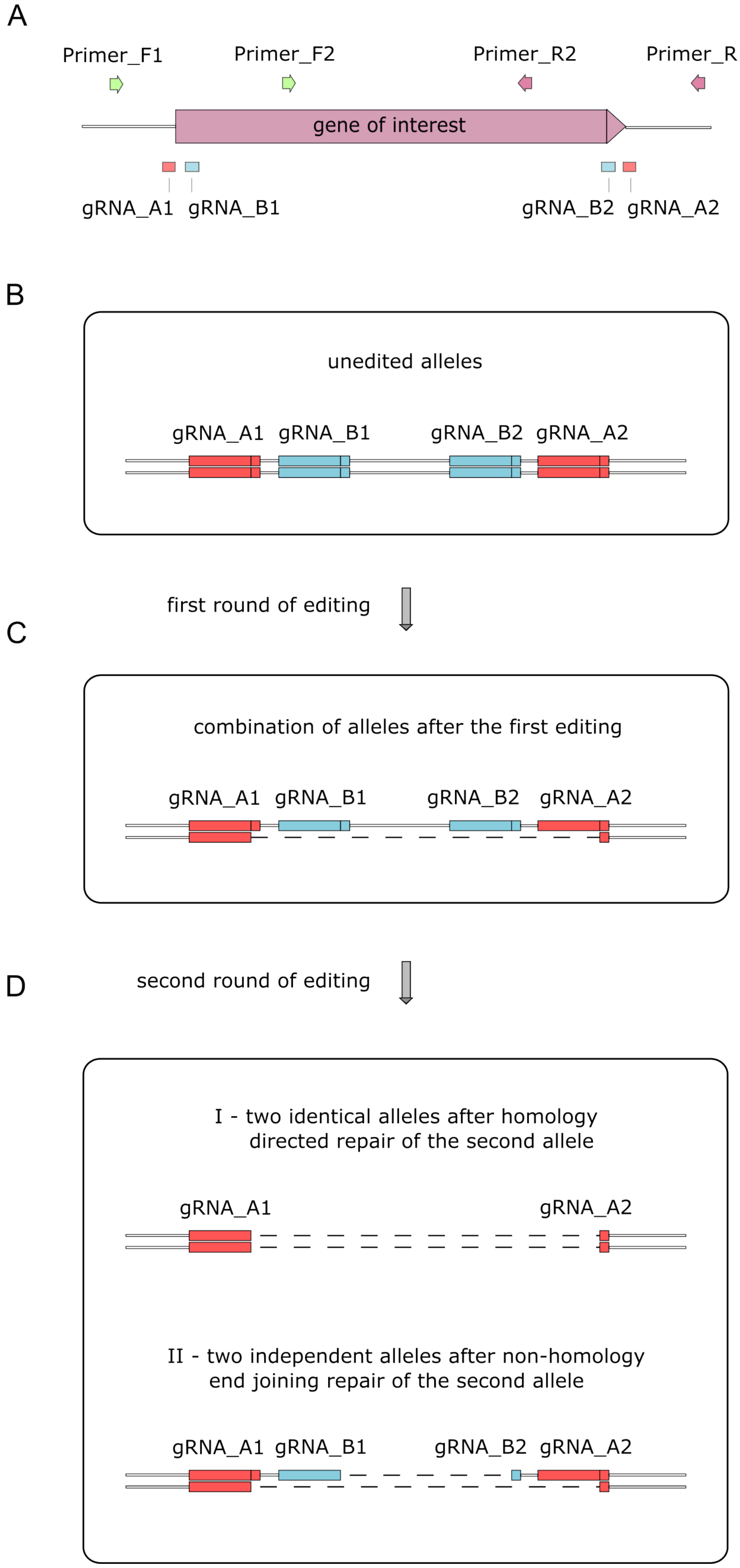

A
Primer_F1
Primer_F2
Primer_R2
Primer_R1
gene of interest
gRNA_A1
gRNA_B1
gRNA_B2
gRNA_A2
B
unedited alleles
gRNA_A1
gRNA_B1
gRNA_B2
gRNA_A2
first round of editing
C
combination of alleles after the first editing
gRNA_A1
gRNA_B1
gRNA_B2
gRNA_A2
D
second round of editing
I - two identical alleles after homology directed repair of the second allele
gRNA_A1
gRNA_A2
II - two independent alleles after non-homology end joining repair of the second allele
gRNA_A1
gRNA_B1
gRNA_B2
gRNA_A2

Figure S13. CRISPR/Cas9-mediated gene editing in cultured *Drosophila* cells. A. Schematics of a gene to be edited with locations of target sequences for gRNAs and primers for genotyping. gRNA_A1 and gRNA_A2 are used for the first round of editing, gRNA_B1 and gRNA_B2 are used for the second round of editing. Primer_F1 and Primer_R1 are used for amplification of the junction after DNA repair and sequencing. Primer_F2 and Primer_R2 are used to screen for the clones with homozygous deletion; no PCR product is expected in homozygous cells. **B.** The schematic of unedited alleles. Rectangles correspond to the position of target sequences for gRNAs, vertical lines inside each rectangle indicate the position of the Cas9 cut in case of the precise deletion. **C.** Combination of alleles in heterozygous cells after the first round of editing. Most of the selected single cell clones bear only one allele with a deletion. **D.** The two most frequent combinations of alleles in the resulting clones after the second round of editing.

Figure S14

A

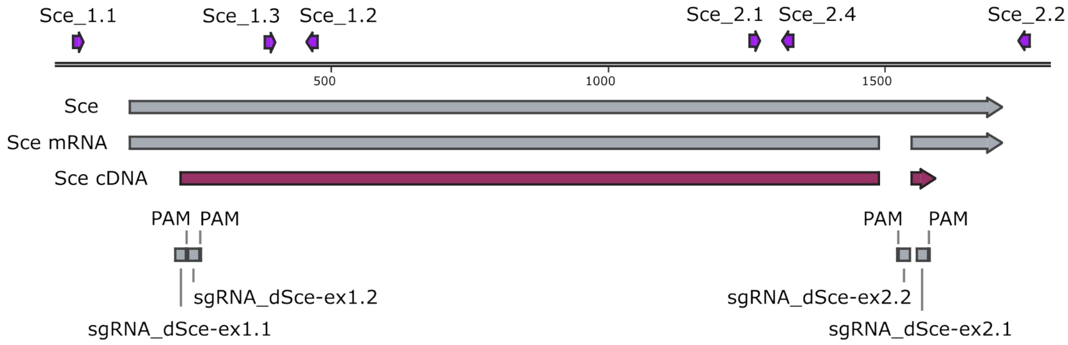

B

DNA sequence structure at the site of editing

Wild type homozygous clone

5'-GAATTGGTGTGAAAATGACGTCGC**TGG**ACCCGGCGCCAAACAAAACGT**GGG**AGCT/-/ATGTTCA**CCA**TTCTAATGTCTGTATGCTTTTAGGTAAACAAACCTATGGAAATGTATTACTCG**TGG**AAG-3'

5'-GAATTGGTGTGAAAATGACGTCGC**TGG**ACCCGGCGCCAAACAAAACGT**GGG**AGCT/-/ATGTTCA**CCA**TTCTAATGTCTGTATGCTTTTAGGTAAACAAACCTATGGAAATGTATTACTCG**TGG**AAG-3'

sgRNA_dSce-ex1.1 sgRNA_dSce-ex1.2 sgRNA_dSce-ex2.2 sgRNA_dSce-ex2.1

Heterozygous clone Sce-63-2

5'-GAATTGGTGTGAAAATGACGTCGC**TGG**ACCCGGCGCCAAACAAAACGT**GGG**AGCT/-/ATGTTCA**CCA**TTCTAATGTCTGTATGCTTTTAGGTAAACAAACCTATGGAAATGTATTACTCG**TGG**AAG-3'

5'-GAATTGGTGTGAAAATGACGT---TCG**TGG**AAG-3'

Homozygous clone Sce-KO-A

5'-GAATTGGTGTGAAAATGACGT---TCG**TGG**AAG-3'

5'-GAATTGGTGTGAAAATGACGT---TCG**TGG**AAG-3'

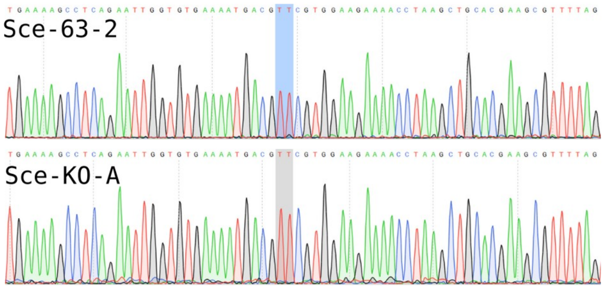

Heterozygous clone Sce-66

5'-GAATTGGTGTGAAAATGACGTCGC**TGG**ACCCGGCGCCAAACAAAACGT**GGG**AGCT/-/ATGTTCA**CCA**TTCTAATGTCTGTATGCTTTTAGGTAAACAAACCTATGGAAATGTATTACTCG**TGG**AAGAAAACCTAAGC-3'

5'-GAATTGGTGTGAAAATGAC---CTAAGC-3'

Homozygous clone Sce-KO-B

5'-GAATTGGTGTGAAAATGAC---CTAAGC-3'

5'-GAATTGGTGTGAAAATGAC---CTAAGC-3'

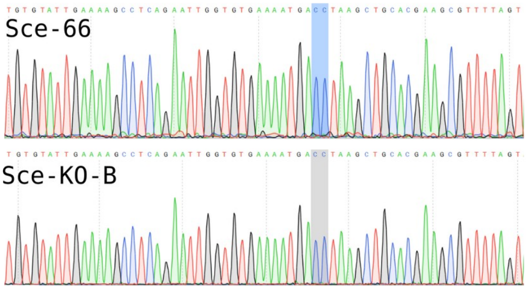

Figure S14. Generation of cultured RING1-KO Drosophila cells. A. The *Sce* gene structure and location of target nucleotide sequences for guide RNAs and primers. The *Sce* gene, mRNA and cDNA are represented as long arrows. Primer locations are indicated as pink arrows, locations of target nucleotide sequences for guide RNAs with corresponding Protospacer Adjacent Motifs (PAMs) are shown as grey boxes. The nucleotide sequences of PCR primers are listed in Table S3. **B.** DNA sequences of both homologous chromosomes at the editing site in unedited cells, heterozygous cells and homozygous cells from the resulting cell lines. The gRNA target sequences are shown in green for the outer pair of gRNAs, in blue for the inner pair of gRNAs. Triplets in bold correspond to PAMs. Red dashed lines mark the position of deleted nucleotides. Blue and grey vertical shadows on sequencing chromatograms mark the position of the junction.

Table S1. Correlation of the ChIP signals between replicate experiments

Sample ID	Sample name	Antibody	File name	Spearman correlation
S9	SceA7-3-H2Aub	H2AK118ub	normalized_signal_SceA7-3-H2Aub_S229_q30_paired_50bp_bins_mean.bed_updated.bedgraph	
S10	SceA7-4-H2Aub	H2AK118ub	normalized_signal_SceA7-4-H2Aub_S230_q30_paired_50bp_bins_mean.bed_updated.bedgraph	0.735
S11	L10-8-H2Aub	H2AK118ub	normalized_signal_L10-8-H2Aub_S231_q30_paired_50bp_bins_mean.bed_updated.bedgraph	
S12	L10-9-H2Aub	H2AK118ub	normalized_signal_L10-9-H2Aub_S232_q30_paired_50bp_bins_mean.bed_updated.bedgraph	0.897
S13	Psc4-15-H2Aub	H2AK118ub	normalized_signal_Psc4-15-H2Aub_S233_q30_paired_50bp_bins_mean.bed_updated.bedgraph	
S14	Psc4-16-H2Aub	H2AK118ub	normalized_signal_Psc4-16-H2Aub_S234_q30_paired_50bp_bins_mean.bed_updated.bedgraph	0.910
S15	Ras17-6-H2Aub	H2AK118ub	normalized_signal_Ras17-6-H2Aub_S235_q30_paired_50bp_bins_mean.bed_updated.bedgraph	
S16	Ras17-7-H2Aub	H2AK118ub	normalized_signal_Ras17-7-H2Aub_S236_q30_paired_50bp_bins_mean.bed_updated.bedgraph	0.898
S19	Ras17-6-RING	RING1	normalized_signal_Ras17-6-RING_S239_q30_paired_50bp_bins_mean.bed_updated.bedgraph	
S20	Ras17-7-RING	RING1	normalized_signal_Ras17-7-RING_S240_q30_paired_50bp_bins_mean.bed_updated.bedgraph	0.736
S21	Ras17-6-Psc	Psc-AS	normalized_signal_Ras17-6-Psc_S241_q30_paired_50bp_bins_mean.bed_updated.bedgraph	
S22	Ras17-7-Psc	Psc-AS	normalized_signal_Ras17-7-Psc_S242_q30_paired_50bp_bins_mean.bed_updated.bedgraph	0.766
S23	Ras17-6-H3K27me3	H3K27me3	normalized_signal_Ras17-6-H3K27me3_S243_q30_paired_50bp_bins_mean.bed_updated.bedgraph	
S24	Ras17-7-H3K27me3	H3K27me3	normalized_signal_Ras17-7-H3K27me3_S244_q30_paired_50bp_bins_mean.bed_updated.bedgraph	0.900
S17	SceA7-3-RING	RING1	normalized_signal_SceA7-3-RING_S237_q30_paired_50bp_bins_mean.bed_updated.bedgraph	
S18	SceA7-4-RING	RING1	normalized_signal_SceA7-4-RING_S237_q30_paired_50bp_bins_mean.bed_updated.bedgraph	0.767

Table S2. The list of antibodies and affinity reagents used in the study

Antigen	Host	Provider/catalogue #/PubMed ID	IF	Western	ChIP
H3	Rabbit polyclonal	Abcam, #ab1791		1:100000	
H2AK119ub	Rabbit monoclonal	Cell Signaling Technology, #8240		1:5000	0.5μl
H3K27me3	Rabbit polyclonal	Abcam, #ab6002		1:2000	0.5μl
RING1	Rabbit polyclonal	this study, Agrisera, #AS163829			1μg
Psc	Rabbit polyclonal	this study, Agrisera, #AS174129			1.5μg
Cp190	Mouse monoclonal	H.Saumweber, clone Bx63, PMID:3143740			2 μl
RING1	Rabbit polyclonal	SDI, Q3200		1:4000	
Psc	Rabbit polyclonal	V. Pirrotta, PMID:11581156		1:1000	
Pc	Rabbit polyclonal	V. Pirrotta, PMID:11581156		1:2000	
H3K27me2	Rabbit polyclonal	Cell Signaling Technology, #9728		1:2000	
StrepTactin sepharose		IBA Lifesciences, #2-1206-002			40μl
Anti-StrepTactin AP conjugated		IBA Lifesciences, #2-1503-001		1:4000	
Anti-rabbit AP conjugated	Goat polyclonal	Promega, #S3731		1:10000	
Abd-B	Mouse monoclonal	DHSB, 1A2E9	1:10		
Antp	mouse monoclonal	DSHB, Antp 4C3	1:50		
Prospero	mouse monoclonal	DSHB, MR1A	1:50		
Anti-mouse Alexa-555	Goat	Abcam, #ab150114	1:300		
Anti-rabbit Alexa-488	Goat	Invitrogen, #A-11008	1:300		
GFP	Rabbit polyclonal	Abcam, #ab290	1:50		
GFP A11122	Rabbit polyclonal	Thermofisher, A-11122	1:600		

Table S3. The list of PCR primers for ChIP-qPCR, RT-qPCR and Sce deletion genotyping

Amplicon name for ChIP-qPCR	Primer name	5'-3' sequence
Control	3L-nc-1.1	AAACTGCCGCACGACGGAGG
Control	3L-nc-1.2	CGGCCCTGCAACGCCTGTAA
62D	62D-1.1	ACTTTTGGGCTGCACGTATC
62D	62D-1.2	GCGCGGGACAAACTTATTTA
Fub	Fub-1.1	AAGGACTCGCTCATGCCTAA
Fub	Fub-1.2	CTTTCGGCAGGTAGCTCAAG
CP1901M	CP1901Mfw	TATTCTGCGCTATGCCTCTTG
CP1901M	CP1901Mrev	TTGGAGTCTGCGCTATGTTC
Homie	Homie1	TATTGTAGGGACAGGTTGATGG
Homie	Homie2	CAGCATACTTTTCGGCTATGTC
BC1M	BC1Mfw	CGCTAACAGGGAGGATGTATTC
BC1M	BC1Mrev	GGCTTGCAAAAATCCTATTTGA
bxb	BP3	GCCATAACGGCAGAACCAAAG
bxb	BP4	ATGAGGCCATCTCAGTCGC
bx	Bx1.1	AACACATACGCGGCACCTAC
bx	Bx1.2	AAATAACCCAGCCCCGTTCT
Abd-B	AbdB-PRE_2.1	CACGACGGAACTCACCTCAG
Abd-B	AbdB-PRE_2.2	CTCACTTGACGATCGCCGTA
oc	oc-1.1	CTACCGCGCGCCAGAGTGAG
oc	oc-1.2	AGTCAGGTTGTAAGCGGCGTGC
disco	disco-1.1	CGCTGCTGCGACGTCACTTT
disco	disco-1.2	TCGTGCGGGTGCGATAGGGA
salm	salm-1.1	TCCTTCCGCCTTTTGCGGCTC
salm	salm-1.2	GGCTCGTTGGCGGGGGAAG
**Amplicon name for RT-qPCR**	**Primer name**	**5'-3' sequence**
Sce	Sce_1.3	AGACGATGACGACGAAGGAGT
Sce	Sce_1.2	GGCACTCCTTATTGCCCGAA
Psc	PSCEX1.1	TCCATTGACCATTCGCACAG
Psc	PSCEX1.2	TTTCACCTTGATGGGTTTCAG
Su(z)2	Su(z)2-ex1.1	GCCAAGGCCCAGAGCTACGC
Su(z)2	Su(z)2-ex1.2	GGTGGTCCCATCAAGCGGGC
sist	sist_ex3_1.1	AGAAGGTGATGGACGCCCACG
sist	sist_ex3_1.2	GCACTCCAGGCACACGTTCAC
RpL32	RpL32-ex1.1	TGGGCGATCTCGCCGCAGTA
RpL32	RpL32-ex1.2	CAGAGTGCGTCGCCGCTTCA
**Amplicon name for Sce deletion genotyping**	**Primer name**	**5'-3' sequence**
Sce_1.1	Sce_1.1	GCGCCAAGCTACGTACTAAA
Sce_1.2	Sce_1.2	GGCACTCCTTATTGCCCGAA
Sce_2.1	Sce_2.1	GCTCATACGGGCACTCAAGG
Sce_2.2	Sce_2.2	CTGTGGACTTATAGCGAGGGA
Sce_2.4	Sce_2.4	TGCTGAGATGATCGACGGTG
Sce_1.3	Sce_1.3	AGACGATGACGACGAAGGAGT

Table S4. Fractions used to prepare sequencing libraries.

Sample ID	Sample name	Antibody	Fraction of IP or input taken for library prep	Fraction of libray taken to 10nM pool
S1	SceA7-3-Input	Input	0.0048	0.102
S2	SceA7-4-Input	Input	0.0156	0.040
S3	L10-8-Input	Input	0.0061	0.031
S4	L10-9-Input	Input	0.0065	0.028
S5	Psc4-15-Input	Input	0.0060	0.034
S6	Psc4-16-Input	Input	0.0056	0.036
S7	Ras17-6-Input	Input	0.0070	0.039
S8	Ras17-7-Input	Input	0.0080	0.039
S9	SceA7-3-H2Aub	H2AK118ub	0.65	0.183
S10	SceA7-4-H2Aub	H2AK118ub	0.65	0.179
S11	L10-8-H2Aub	H2AK118ub	0.25	0.095
S12	L10-9-H2Aub	H2AK118ub	0.25	0.068
S13	Psc4-15-H2Aub	H2AK118ub	0.25	0.046
S14	Psc4-16-H2Aub	H2AK118ub	0.25	0.057
S15	Ras17-6-H2Aub	H2AK118ub	0.175	0.034
S16	Ras17-7-H2Aub	H2AK118ub	0.125	0.045
S17	SceA7-3-RING	RING1	0.65	0.160
S18	SceA7-4-RING	RING1	0.65	0.124
S19	Ras17-6-RING	RING1	0.5	0.096
S20	Ras17-7-RING	RING1	0.5	0.111
S21	Ras17-6-Psc	Psc	0.325	0.196
S22	Ras17-7-Psc	Psc	0.45	0.113
S23	Ras17-6-H3K27me3	H3K27me3	0.35	0.102
S24	Ras17-7-H3K27me3	H3K27me3	0.275	0.094

Table S5. The list of primers and amplification conditions for the preparation of mapping TRIP libraries

Primer name	5'-3' sequence	PCR amplification round	Tann, C	Annealing time, sec	Extention time, min	No of cycles
PB-outer-F-2	TTTTACGCATGATTATCTTTAACGTACGTC					
cDNA-ampl-R	CGCCAGGGTTTTCCCAGTCACAAG	1	62	30	2.5	12
PB-cDNA-fwd-mod1-AN*	TCGTCGGCAGCGTCAGATGTGTATAAGAGACAGNNNNNNNNGTCACAAGGGCTGGCCACAA					
InvPCR-F-Nextera2	GTCTCGTGGGCTCGGAGATGTGTATAAGAGACAGGTACGTCACAATATGATTATCTTTCTAG	2	61	30	2.5	12
Libr-P5-for	AATGATACGGCGACCACCGAGATCTACACTCGTCGGCAGCGTC					
Libr-P7-rev	CAAGCAGAAGACGGCATACGAGATGTCTCGTGGGCTCGG	3	66	30	2.5	12

* Variants of PB-cDNA-fwd-mod1-AN primer listed below were used to prepare different libraries

PB-cDNA-fwd-mod1-A1	TCGTCGGCAGCGTCAGATGTGTATAAGAGACAGTTCGGAGTGTCACAAGGGCTGGCCACAA
PB-cDNA-fwd-mod1-A2	TCGTCGGCAGCGTCAGATGTGTATAAGAGACAGACTCATTTGTCACAAGGGCTGGCCACAA
PB-cDNA-fwd-mod1-A3	TCGTCGGCAGCGTCAGATGTGTATAAGAGACAGGGGATCCGGTCACAAGGGCTGGCCACAA
PB-cDNA-fwd-A5	TCGTCGGCAGCGTCAGATGTGTATAAGAGACAGCAAGATAAGTCACAAGGGCTGGCCACAA
PB-cDNA-fwd-A6	TCGTCGGCAGCGTCAGATGTGTATAAGAGACAGGGACAACGGTCACAAGGGCTGGCCACAA
PB-cDNA-fwd-A7	TCGTCGGCAGCGTCAGATGTGTATAAGAGACAGAGCGAGCTGTCACAAGGGCTGGCCACAA
PB-cDNA-fwd-A8	TCGTCGGCAGCGTCAGATGTGTATAAGAGACAGCTGCACGTGTCACAAGGGCTGGCCACAA
PB-cDNA-fwd-A10	TCGTCGGCAGCGTCAGATGTGTATAAGAGACAGAGTCGCCGGTCACAAGGGCTGGCCACAA

Table S6. The list of sequence libraries produced for TRIP experiment

TRIP library name	Sample name	Raw .fastq file name	demultiplexed .fastq files (trip_bash_awk.sh)	Illumina sequencing index	Type of the sample	Index primer name	Cell line	Treatment	Replicate	Source for ChIP-seq library preparation
Sce-A1	Sce-KO-M1	Sce-M1_1.fastq, Sce-M1_2.fastq		TTCGGAGT	TRIP-mapping library	PB-cDNA-fwd-mod1-A1	Ras17-Sce-KO-TRIP-pBacH1-10k		1	inverse PCR product
Sce-A6	Sce-KO-M2	Sce-M2_1.fastq, Sce-M2_2.fastq		GGACAACG	TRIP-mapping library	PB-cDNA-fwd-A6	Ras17-Sce-KO-TRIP-pBacH1-10k		2	inverse PCR product
Psc-A2	Psc-Su(z)2-KO-M1	Psc-M1_1.fastq, Psc-M1_2.fastq		ACTCATTT	TRIP-mapping library	PB-cDNA-fwd-mod1-A2	Psc-KO-TRIP-pBacH1-10k		1	inverse PCR product
Psc-A7	Psc-Su(z)2-KO-M2	Psc-M2_1.fastq, Psc-M2_2.fastq		AGCGAGCT	TRIP-mapping library	PB-cDNA-fwd-A7	Psc-KO-TRIP-pBacH1-10k		2	inverse PCR product
L10-A3	Sist-KO-M1	L10-P28-M1_1.fastq, L10-P28-M1_2.fastq		GGGATCCG	TRIP-mapping library	PB-cDNA-fwd-mod1-A3	Sist-KO-TRIP-pBacH1-10k		1	inverse PCR product
L10-A8	Sist-KO-M2	L10-P29-M2_1.fastq, L10-P29-M2_2.fastq		CTGCACGT	TRIP-mapping library	PB-cDNA-fwd-A8	Sist-KO-TRIP-pBacH1-10k		2	inverse PCR product
Ras17-T1-A5	Ras17-M1	Ras17-M1_1.fastq, Ras17-M1_2.fastq		CAAGATAA	TRIP-mapping library	PB-cDNA-fwd-A5	Ras17-TRIP-pBacH1-10k		1	inverse PCR product
Ras17-T2-A10	Ras17-M2	Ras17-M2_1.fastq, Ras17-M2_2.fastq		AGTCGCCG	TRIP-mapping library	PB-cDNA-fwd-A10	Ras17-TRIP-pBacH1-10k		2	inverse PCR product
Sce-N-A12	Sce-KO-N1	Sce-N1_1.fastq, Sce-N1_2.fastq	Sce-N1_1_11.fastq, Sce-N1_1_3.fastq, Sce-N1_1_16.fastq, Sce-N1_1_14.fastq, Sce-N1_2_11.fastq, Sce-N1_2_3.fastq, Sce-N1_2_16.fastq, Sce-N1_2_14.fastq	ACAATTCG	TRIP-normalization library	Libr-cDNA-A12-rev	Ras17-Sce-KO-TRIP-pBacH1-10k		1	genomic DNA
Psc-N-A13	Psc-Su(z)2-KO-N1	Psc-N1_1.fastq, Psc-N1_2.fastq	Psc-N1_1_11.fastq, Psc-N1_1_3.fastq, Psc-N1_1_16.fastq, Psc-N1_1_14.fastq, Psc-N1_2_11.fastq, Psc-N1_2_3.fastq, Psc-N1_2_16.fastq, Psc-N1_2_14.fastq	TACTTGTC	TRIP-normalization library	Libr-cDNA-A13-rev	Psc-KO-TRIP-pBacH1-10k		1	genomic DNA
L10-N-A14	Sist-KO-N1	L10-N1_1.fastq, L10-N1_2.fastq	L10-N1_1_11.fastq, L10-N1_1_3.fastq, L10-N1_1_14.fastq, L10-N1_1_16.fastq, L10-N1_2_11.fastq, L10-N1_2_3.fastq, L10-N1_2_14.fastq, L10-N1_2_16.fastq	GTACCGTT	TRIP-normalization library	Libr-cDNA-A14-rev	Sist-KO-TRIP-pBacH1-10k		1	genomic DNA
Ras17-N-A15	Ras17-N1	Ras17-N1_1.fastq, Ras17-N1_2.fastq	Ras17-N1_1_11.fastq, Ras17-N1_1_3.fastq, Ras17-N1_1_16.fastq, Ras17-N1_1_14.fastq, Ras17-N1_2_11.fastq, Ras17-N1_2_3.fastq, Ras17-N1_2_16.fastq, Ras17-N1_2_14.fastq	GCCACATA	TRIP-normalization library	Libr-cDNA-A15-rev	Ras17-TRIP-pBacH1-10k		1	genomic DNA
Sce-N-A16	Sce-KO-N2	Sce-N2_1.fastq, Sce-N2_2.fastq,	Sce-N2_1_11.fastq, Sce-N2_1_3.fastq, Sce-N2_1_16.fastq, Sce-N2_1_14.fastq, Sce-N2_2_11.fastq, Sce-N2_2_3.fastq, Sce-N2_2_16.fastq, Sce-N2_2_14.fastq	CCTATGGT	TRIP-normalization library	Libr-cDNA-A16-rev	Ras17-Sce-KO-TRIP-pBacH1-10k		2	genomic DNA
Psc-N-A17	Psc-Su(z)2-KO-N2	Psc-N2_1.fastq, Psc-N2_2.fastq	Psc-N2_1_11.fastq, Psc-N2_1_3.fastq, Psc-N2_1_16.fastq, Psc-N2_1_14.fastq, Psc-N2_2_11.fastq, Psc-N2_2_3.fastq, Psc-N2_2_16.fastq, Psc-N2_2_14.fastq	AACGTCGC	TRIP-normalization library	Libr-cDNA-A17-rev	Psc-KO-TRIP-pBacH1-10k		2	genomic DNA
L10-N-A18	Sist-KO-N2	L10-N2_1.fastq, L10-N2_2.fastq	L10-N2_1_11.fastq, L10-N2_1_3.fastq, L10-N2_1_14.fastq, L10-N2_1_16.fastq, L10-N2_2_11.fastq, L10-N2_2_3.fastq, L10-N2_2_14.fastq, L10-N2_2_16.fastq	AGGCAGCA	TRIP-normalization library	Libr-cDNA-A18-rev	Sist-KO-TRIP-pBacH1-10k		2	genomic DNA
Ras17-N-A19	Ras17-N2	Ras17-N2_1.fastq, Ras17-N2_2.fastq	Ras17-N2_1_11.fastq, Ras17-N2_1_3.fastq, Ras17-N2_1_16.fastq, Ras17-N2_1_14.fastq, Ras17-N2_2_11.fastq, Ras17-N2_2_3.fastq, Ras17-N2_2_16.fastq, Ras17-N2_2_14.fastq	AGCTTTCT	TRIP-normalization library	Libr-cDNA-A19-rev	Ras17-TRIP-pBacH1-10k		2	genomic DNA
Sce-T1-A20	Sce-KO-T1	Sce-T1_1.fastq, Sce-T1_2.fastq		GGTATGTT	TRIP-cDNA library	Libr-cDNA-A20-rev	Ras17-Sce-KO-TRIP-pBacH1-10k		1	cDNA
Psc-T1-A21	Psc-Su(z)2-KO-T1	Psc-T1_1.fastq, Psc-T1_2.fastq		GAGGGACC	TRIP-cDNA library	Libr-cDNA-A21-rev	Psc-KO-TRIP-pBacH1-10k		1	cDNA

L10-T1-A22	Sist-KO-T1	L10-T1_1.fastq, L10-T1_2.fastq		TAGCTCTA	TRIP-cDNA library	Libr-cDNA-A22-rev	Sist-KO-TRIP-pBacH1-10k		1	cDNA
R17-T1-A23	Ras17-T1	R17-T1_1.fastq, R17-T1_2.fastq		TAATTGCG	TRIP-cDNA library	Libr-cDNA-A23-rev	Ras17-TRIP-pBacH1-10k		1	cDNA
Sce-T1-2H-A24	Sce-KO-T1-2H	Sce-T1-2H_1.fastq, Sce-T1-2H_2.fastq		GAAATGGG	TRIP-cDNA library	Libr-cDNA-A24-rev	Ras17-Sce-KO-TRIP-pBacH1-10k	0.5mM CuSO4, 2 hours	1	cDNA
Psc-T1-2H-A25	Psc-Su(z)2-KO-T1-2H	Psc-T1-2H_1.fastq, Psc-T1-2H_2.fastq		TCGAGACT	TRIP-cDNA library	Libr-cDNA-A25-rev	Psc-KO-TRIP-pBacH1-10k	0.5mM CuSO4, 2 hours	1	cDNA
L10-T1-2H-A26	Sist-KO-T1-2H	L10-T1-2H_1.fastq, L10-T1-2H_2.fastq		CAGAGAGG	TRIP-cDNA library	Libr-cDNA-A26-rev	Sist-KO-TRIP-pBacH1-10k	0.5mM CuSO4, 2 hours	1	cDNA
R17-T1-2H-A27	Ras17-T1-2H	R17-T1-2H_1.fastq, R17-T1-2H_2.fastq		ATTAGTCA	TRIP-cDNA library	Libr-cDNA-A27-rev	Ras17-TRIP-pBacH1-10k	0.5mM CuSO4, 2 hours	1	cDNA
Sce-T2-A28	Sce-KO-T2	Sce-T2_1.fastq, Sce-T2_2.fastq		CTAAGGCC	TRIP-cDNA library	Libr-cDNA-A28-rev	Ras17-Sce-KO-TRIP-pBacH1-10k		2	cDNA
Psc-T2-A29	Psc-Su(z)2-KO-T2	Psc-T2_1.fastq, Psc-T2_2.fastq		GTATACCG	TRIP-cDNA library	Libr-cDNA-A29-rev	Psc-KO-TRIP-pBacH1-10k		2	cDNA
L10-T2-A30	Sist-KO-T2	L10-T2_1.fastq, L10-T2_2.fastq		CCTTCTGA	TRIP-cDNA library	Libr-cDNA-A30-rev	Sist-KO-TRIP-pBacH1-10k		2	cDNA
R17-T2-A31	Ras17-T2	R17-T2_1.fastq, R17-T2_2.fastq		TTGGCGAG	TRIP-cDNA library	Libr-cDNA-A31-rev	Ras17-TRIP-pBacH1-10k		2	cDNA
Sce-T2-2H-A32	Sce-KO-T2-2H	Sce-T2-2H_1.fastq, Sce-T2-2H_2.fastq		TAGAAGAG	TRIP-cDNA library	Libr-cDNA-A32-rev	Ras17-Sce-KO-TRIP-pBacH1-10k	0.5mM CuSO4, 2 hours	2	cDNA
Psc-T2-2H-A33	Psc-Su(z)2-KO-T2-2H	Psc-T2-2H_1.fastq, Psc-T2-2H_2.fastq		TGACAGAT	TRIP-cDNA library	Libr-cDNA-A33-rev	Psc-KO-TRIP-pBacH1-10k	0.5mM CuSO4, 2 hours	2	cDNA
L10-T2-2H-A34	Sist-KO-T2-2H	L10-T2-2H_1.fastq, L10-T2-2H_2.fastq		ACATTTAC	TRIP-cDNA library	Libr-cDNA-A34-rev	Sist-KO-TRIP-pBacH1-10k	0.5mM CuSO4, 2 hours	2	cDNA
R17-T2-2H-A35	Ras17-T2-2H	R17-T2-2H_1.fastq, R17-T2-2H_2.fastq		GATTAACC	TRIP-cDNA library	Libr-cDNA-A35-rev	Ras17-TRIP-pBacH1-10k	0.5mM CuSO4, 2 hours	2	cDNA

Table S7. The list of primers and amplification conditions for the preparation of normalization and expression TRIP libraries

Primer name	5'-3' sequence	PCR amplification round	Tann, C	Extention time, seconds	No of cycles
Libr-cDNA-for	GTCTCGTGGGCTCGGAGATGTGTATAAGAGACAGGTCCTGCTGGAGTTCGTGAC				
Libr-cDNA-AN-rev*	TCGTCGGCAGCGTCAGATGTGTATAAGAGACAGNNNNNNNNCGCCAGGGTTTTCCCAGTCACAAGG	1	72	20	15
Libr-P5-for	AATGATACGGCGACCACCGAGATCTACACTCGTCGGCAGCGTC				
Libr-P7-rev	CAAGCAGAAGACGGCATACGAGATGTCTCGTGGGCTCGG	2	72	20	15

* Variants of Libr-cDNA-AN-rev primer listed below were used to prepare different libraries

Libr-cDNA-A12-rev	TCGTCGGCAGCGTCAGATGTGTATAAGAGACAGACAATTCGCGCCAGGGTTTTCCCAGTCACAAGG
Libr-cDNA-A13-rev	TCGTCGGCAGCGTCAGATGTGTATAAGAGACAGTACTTGTCCGCCAGGGTTTTCCCAGTCACAAGG
Libr-cDNA-A14-rev	TCGTCGGCAGCGTCAGATGTGTATAAGAGACAGGTACCGTTCGCCAGGGTTTTCCCAGTCACAAGG
Libr-cDNA-A15-rev	TCGTCGGCAGCGTCAGATGTGTATAAGAGACAGGCCACATACGCCAGGGTTTTCCCAGTCACAAGG
Libr-cDNA-A16-rev	TCGTCGGCAGCGTCAGATGTGTATAAGAGACAGCCTATGGTCGCCAGGGTTTTCCCAGTCACAAGG
Libr-cDNA-A17-rev	TCGTCGGCAGCGTCAGATGTGTATAAGAGACAGAACGTCGCCGCCAGGGTTTTCCCAGTCACAAGG
Libr-cDNA-A18-rev	TCGTCGGCAGCGTCAGATGTGTATAAGAGACAGAGGCAGCACGCCAGGGTTTTCCCAGTCACAAGG
Libr-cDNA-A19-rev	TCGTCGGCAGCGTCAGATGTGTATAAGAGACAGAGCTTTCTCGCCAGGGTTTTCCCAGTCACAAGG
Libr-cDNA-A20-rev	TCGTCGGCAGCGTCAGATGTGTATAAGAGACAGGGTATGTTCGCCAGGGTTTTCCCAGTCACAAGG
Libr-cDNA-A21-rev	TCGTCGGCAGCGTCAGATGTGTATAAGAGACAGGAGGGACCCGCCAGGGTTTTCCCAGTCACAAGG
Libr-cDNA-A22-rev	TCGTCGGCAGCGTCAGATGTGTATAAGAGACAGTAGCTCTACGCCAGGGTTTTCCCAGTCACAAGG
Libr-cDNA-A23-rev	TCGTCGGCAGCGTCAGATGTGTATAAGAGACAGTAATTGCGCGCCAGGGTTTTCCCAGTCACAAGG
Libr-cDNA-A24-rev	TCGTCGGCAGCGTCAGATGTGTATAAGAGACAGGAAATGGGCGCCAGGGTTTTCCCAGTCACAAGG
Libr-cDNA-A25-rev	TCGTCGGCAGCGTCAGATGTGTATAAGAGACAGTCGAGACTCGCCAGGGTTTTCCCAGTCACAAGG
Libr-cDNA-A26-rev	TCGTCGGCAGCGTCAGATGTGTATAAGAGACAGCAGAGAGGCGCCAGGGTTTTCCCAGTCACAAGG
Libr-cDNA-A27-rev	TCGTCGGCAGCGTCAGATGTGTATAAGAGACAGATTAGTCACGCCAGGGTTTTCCCAGTCACAAGG
Libr-cDNA-A28-rev	TCGTCGGCAGCGTCAGATGTGTATAAGAGACAGCTAAGGCCCGCCAGGGTTTTCCCAGTCACAAGG
Libr-cDNA-A29-rev	TCGTCGGCAGCGTCAGATGTGTATAAGAGACAGGTATACCGCGCCAGGGTTTTCCCAGTCACAAGG
Libr-cDNA-A30-rev	TCGTCGGCAGCGTCAGATGTGTATAAGAGACAGCCTTCTGACGCCAGGGTTTTCCCAGTCACAAGG
Libr-cDNA-A31-rev	TCGTCGGCAGCGTCAGATGTGTATAAGAGACAGTTGGCGAGCGCCAGGGTTTTCCCAGTCACAAGG
Libr-cDNA-A32-rev	TCGTCGGCAGCGTCAGATGTGTATAAGAGACAGTAGAAGAGCGCCAGGGTTTTCCCAGTCACAAGG
Libr-cDNA-A33-rev	TCGTCGGCAGCGTCAGATGTGTATAAGAGACAGTGACAGATCGCCAGGGTTTTCCCAGTCACAAGG
Libr-cDNA-A34-rev	TCGTCGGCAGCGTCAGATGTGTATAAGAGACAGACATTTACCGCCAGGGTTTTCCCAGTCACAAGG
Libr-cDNA-A35-rev	TCGTCGGCAGCGTCAGATGTGTATAAGAGACAGGATTAACCCGCCAGGGTTTTCCCAGTCACAAGG

Supplementary movie 1. Locomotion of the control first instar larvae under normal (21%) oxygen level.

Supplementary movie 2. Locomotion of the Siesta-KO^{mz} first instar larvae under normal (21%) oxygen level.

Supplementary movie 3. Locomotion of the control first instar larvae under variable oxygen levels.

Supplementary movie 4. Locomotion of the Siesta-KO^{mz} first instar larvae under variable oxygen levels.

Supplementary file 1. Genome segmentation into 1kb windows with associated ChIP-seq signal values and cluster assignments.

Supplementary file 2. Annotated DNA sequence of the pPB-MtnA-eGFP-PI-14 construct.

Supplementary file 3. Annotated DNA sequence of the pPB-MtnA-eGFP-PI-16 construct.

Supplementary file 4. Annotated DNA sequence of the pPB-Hsp70-eGFP-PI-11 construct.

Supplementary file 5. Annotated DNA sequence of the pPB-Hsp70-eGFP-PI-3 construct.

Supplementary file 6. Annotated DNA sequence of the phsp70-H1-L1-pBac construct.

Supplementary file 7. A bash script for demultiplexing of TRIP data.

Supplementary file 8. Configuration file for TRIP analysis with TRIP Analysis Software Kit (TASK).

Supplementary file 9. The example command line instructions to process a dataset with TASK software.

Supplementary file 10. The list of genuine transgenic insertions with associated barcode sequences, genomic positions and TRIP signals.
